# Endogenous and exogenous control of visuospatial selective attention in freely behaving mice

**DOI:** 10.1038/s41467-020-15909-2

**Published:** 2020-04-24

**Authors:** Wen-Kai You, Shreesh P. Mysore

**Affiliations:** 1 The Solomon H. Snyder Department of Neuroscience, Johns Hopkins University, Baltimore, MD 21218 USA; 2 Department of Psychological and Brain Sciences, Johns Hopkins University, Baltimore, MD 21218 USA

**Keywords:** Attention, Sensorimotor processing, Visual system

## Abstract

Visuospatial selective attention has been investigated primarily in head-fixed animals and almost exclusively in primates. Here, we develop two human-inspired, discrimination-based behavioral paradigms for studying selective visuospatial attention in freely behaving mice. In the ‘spatial probability’ task, we find enhanced accuracy, sensitivity, and rate of evidence accumulation at the location with higher probability of target occurrence, and opposite effects at the lower probability location. Together with video-based 3D head-tracking, these results demonstrate endogenous expectation-driven shifts of spatial attention. In the ‘flanker’ task, we find that a second stimulus presented with the target, but with conflicting information, causes switch-like decrements in accuracy and sensitivity as a function of its contrast, and slower evidence accumulation, demonstrating exogenous capture of spatial attention. The ability to study primate-like selective attention rigorously in unrestrained mice opens a rich avenue for research into neural circuit mechanisms underlying this critical executive function in a naturalistic setting.

## Abstract (web summary)

The authors describe behavioural tasks for the study of primate-like, endogenous and exogenous control of visuospatial selective attention in freely behaving mice.

## Introduction

Animals have the remarkable ability to preferentially process the most important, or “highest priority”, information in complex environments to guide behavior. Called selective attention, this ability is essential for a range of cognitive functions and adaptive behavior, and its dysfunction is found in diverse psychiatric illnesses, including ADHD and schizophrenia. A rich body of work into visuospatial selective attention has provided insights into the consequences of selective attention to behavior and neural processing^1^, and has identified the involvement of critical fronto-parietal^2,3^ and midbrain^4^ networks in selective attention. However, the neural circuit mechanisms for the control of visuospatial selective attention remain largely open questions.

The dissection of these circuit mechanisms can benefit greatly from cutting-edge approaches for the interrogation of identified subsets of neurons in a genetically tractable animal system. In contrast to this need, visuospatial selective attention has, thus far, been studied nearly exclusively in primates (but see refs. ^5,6^). Whereas the mouse, a genetically powerful animal model, has been used to study visually guided behavior^7–9^, and recently, circuits for cross-modal attention^10,11^, it has been debated whether mice are capable of exhibiting spatially well-resolved visual behaviors that are necessary to unpack the circuit basis of visuospatial attention, with just one recent study reporting success^6^.

Second, because selective attention operates in nature in animals engaging freely with the world, the study of its neural underpinnings can benefit greatly from investigations in unrestrained animals in a behavioral state resembling natural conditions: with intact vestibular feedback cues^12^, as well as coordinated body movements^13–15^. Many reports have highlighted differences in neural representations as a function of the behavioral state of the animal^16–19^, and specifically, in mice, whose nose-pokes and head motion are an integral component of their natural behavior, the use of unrestrained behavioral paradigms may be important. By contrast, selective (visuospatial) attention has been studied almost exclusively in restrained preparations.

In response to these needs, we present, for the first time, two human-inspired behavioral tasks for the study of endogenous as well as exogenous control of visuospatial selective attention in freely behaving mice. Both tasks are touchscreen-based^20,21^, self-paced, and based on a 2-AFC design that dissociates the locus of spatial attention from the locus of behavioral report. Our study adds support to the recent report of behavioral signatures of selective attention in head-fixed mice^6^, and extends it, by demonstrating them in unrestrained mice. In doing so, it accomplishes three critical goals: (a) the demonstration of primate-like (visuospatial) selective attention, (b) in freely behaving animals, and (c) in a species that facilitates the use of diverse genetically based tools for neural interrogation (Supplementary Fig. 1R).

## Results

### Single-stimulus visual discrimination task

All the behavioral tasks in this study involved a touchscreen-based setup^20,21^ (see Methods). A key aspect of the design of our tasks for spatial attention involved the decoupling of the spatial locus of the target of attention from that of the behavioral report. To achieve this, all mice were first trained to generate behavioral responses based on feature information contained in the target stimulus—here, orientation of the target grating—rather than based on its spatial location (Supplementary Fig. 1A). Immediately upon trial initiation (nose-touch at the zeroing cross), a single-oriented grating (“target”) was presented at a fixed location along the vertical midline (see Methods). Mice were rewarded if they responded to a vertically oriented target with a nose-touch to the left response port, and to a horizontally oriented grating with a nose-touch to the right response port.

Mice learned well the response association rule (Supplementary Fig. 1B–D), and there were neither systematic sensory or perceptual biases with respect to grating orientation (Supplementary Fig. 1E, F) nor motor biases with respect to nose-touch location (Supplementary Fig. 1G). With this task as a foundation, we next trained the mice on selective spatial attention tasks.

### Endogenous control of visuospatial-selective attention

**Fig. 1:**
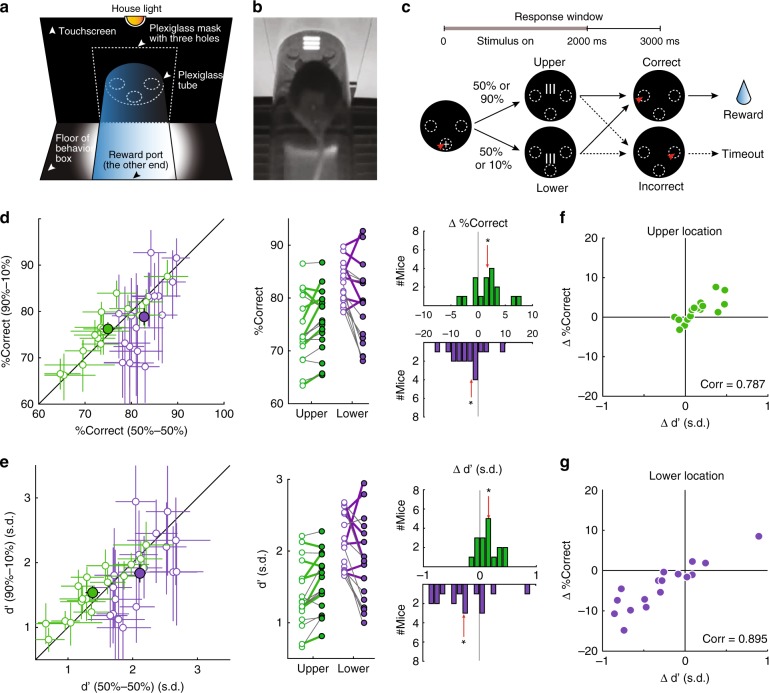
Spatial probability modulates response accuracy and perceptual sensitivity in a space-specific manner (*n* = 17 mice). **a** Schematic touchscreen-based setup. **b** Snapshot of mouse facing a visual stimulus. **c** Schematic of 2-AFC task design. Upper row: trial timeline. Lower row: screenshots of the display at different stages; shown from the mouse’s perspective. Trials began with a nose-touch (red arrowhead) on a zeroing cross within the central hole (dashed oval). A single, oriented grating (target) was presented after trial initiation (see Methods). Grating orientation was either horizontal or vertical; only the vertical grating is shown for simplicity. In the equal probability (50–50) condition, the target was presented with equal probability at the upper or lower locations. In the up-heavy (90u-10) condition, target was presented with 90% probability at the upper location. Mice were rewarded for reporting the orientation of the target grating: vertical → nose-touch to the left; horizontal → nose-touch to the right. The two conditions were run in blocks (one per day), pseudo-randomized across days. **d** Effect of spatial probability on response accuracy. Left panel: scatter plot comparing the performance of mice in two conditions. Each open dot represents one mouse; mean ± 95% confidence intervals shown. Green: upper location, purple: lower location. Large filled dots: group mean values ±  S.E.M. Middle panel: Data replotted to compare mean response accuracy of each mouse between the 50–50 (open dots) and 90u-10 (filled dots) conditions. Colored thick lines connecting dots: mice whose head movements were tracked using an automated video-based system in a subset of sessions (see Supplementary Figs. 3 and 4). Right panel: distributions of change in accuracy (90u-10 condition minus 50–50 condition) derived from middle panel. Upper location: median change = 1.9%, *p* = 0.028; Lower location: median change = −2.5%, *p* = 0.013; red arrow: median; asterisk: *p* < 0.05; two-sided signed-rank and HB tests. **e** Effect of spatial probability on perceptual sensitivity (d’); conventions as in **d**. Upper location: median change = 0.14 s.d., *p* = 0.006; lower location: median change = −0.3 s.d., *p* = 0.028. **f**, **g** Change in accuracy plotted against change in d’ at the upper (**f**, Pearson’s correlation = 0.787, *p* < 0.001) and lower (**g,** Pearson’s correlation = 0.895, *p* < 0.001) locations. See also Supplementary Fig. 1 and Supplementary Movie 1. Source data are provided as a Source Data file.

To study endogenous (top–down) control of visuospatial attention, we trained freely behaving mice on a spatial probability task (Fig. 1c). Previous work in humans has shown that manipulating the spatial probability of target occurrence can serve as an endogenous attentional cue^22–24^. Here, immediately upon trial initiation, a single, oriented grating (“target”) was presented on the screen at one of two locations along the vertical axis in each trial, i.e., at upper or lower location (Fig. 1c). Mice were rewarded for correctly reporting the orientation of the target per the association rule learned previously.

The probability of target occurrence in each trial at the upper versus lower locations was changed in different blocks of trials to manipulate the mouse’s expectation regarding target location. The blocks were of two types: (i) equal probability (or “50–50”) block, in which, on each trial, the target occurred with equal (50%) probability at the upper or the lower locations, and (ii) up-heavy (or “90u–10”) block, in which, on each trial, the target occurred with 90% probability at the upper location, and 10% probability at the lower location (Fig. 1c). Each block lasted for the entire behavioral session on a given day, and blocks of the two types were interleaved pseudo-randomly across days.

In the 90u-10 block, the upper location was chosen to be the higher probability location because pilot experiments revealed an asymmetry in perceptual performance between the two stimulus locations. In the baseline 50–50 condition, response accuracy at the upper location was consistently worse than at the lower location (Supplementary Fig. 1H). Therefore, biasing the probability of target occurrence in favor of the upper location allowed us to test if biased spatial expectation could improve behavioral performance, without any potential confounds due to ceiling effects.

### Effect of spatial probability on response accuracy

To examine the effects of spatial probability of the target on behavioral performance, we started by comparing the response accuracy of mice in the 90u-10 versus the 50–50 blocks for each target location (Fig. 1d). We found that at the upper location, mice (*n* = 17) exhibited a significant improvement in response accuracy in the 90u-10 blocks over that in 50–50 blocks (Fig. 1d; green data, median improvement = 1.9%; *p* = 0.028, signed-rank and HB tests; see Methods). By contrast, at the lower location, mice exhibited a significant worsening of accuracy in the 90u-10 blocks (Fig. 1d, purple data; median reduction = 2.5%; *p* = 0.013, signed-rank and HB test). That is, together a net swing in performance of 4.4% between the upper and lower locations. Thus, spatial probability of the target modulated performance in a spatially selective manner favoring the higher probability location.

Changes in response accuracy can result from changes in the perceptual sensitivity (d’) of animals or their decision criteria (c)^25^: Heightened perceptual sensitivity (d’) improves accuracy, as does reduction in the magnitude of criterion (|c|), whereas reduced d’ or an increase in |c| worsens accuracy (Supplementary Fig. 1J). Because demonstrating improvement in perceptual sensitivity is a critical aspect of the behavioral demonstration of visuospatial-selective attention^26^, we next investigated whether and how spatial probability impacted perceptual sensitivity. Using the standard signal detection theory approach, we computed d’ and |c| at each of the two locations, and for each block type^27^.

We found that at the upper location, animals exhibited an increase in perceptual sensitivity in 90u-10 blocks compared with 50–50 blocks (Fig. 1e; green data; median increase = +0.14 s.d., *p* = 0.006, signed-rank and HB tests; see Methods). By contrast, at the lower location, animals exhibited a significant decrease in perceptual sensitivity (Fig. 1e, purple data; mean decrease = −0.3 s.d., *p* = 0.028, signed-rank and HB tests); a net swing in d’ of 0.44 s.d. between the upper and lower locations (Fig. 1e; right panel). There was no systematic effect of spatial probability on |c|  at either location (Supplementary Fig. 1K, upper location (green data): *p* = 0.981; lower location (purple data): *p* = 0.332, signed-rank and HB tests). Indeed, at both locations, changes in accuracy correlated well with changes in perceptual sensitivity (Fig. 1f, upper location, Pearson correlation coefficient = 0.787, *p* < 0.001; Fig. 1g, lower location, Pearson correlation coefficient = 0.895, *p* < 0.001), but not with changes in |c| (Supplementary Fig. 1L, upper location, green data: Pearson correlation coefficient = −0.271, *p* = 0.292; lower location, purple data: Pearson correlation coefficient = −0.140, *p* = 0.592), indicating that changes in sensitivity, but not criterion, best accounted for the effects on accuracy. Thus, changes to spatial probability of the target also modulated perceptual sensitivity of mice in a spatially selective manner, favoring the higher probability location.

### Effect of spatial probability on RTs

**Fig. 2:**
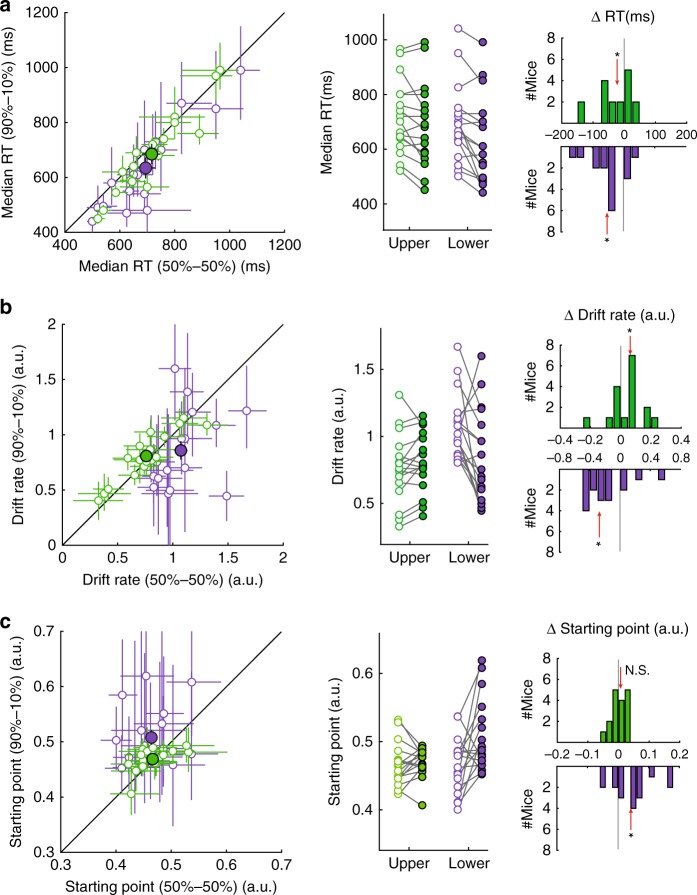
Spatial probability modulates rate of sensory evidence accumulation in a space-specific manner (*n* = 17 mice). **a** Effect of spatial probability on median reaction time (RT). Left panel: scatter plot comparing the median RT of mice in the 90u-10 condition against the 50–50 condition. Each open dot represents one mouse; mean ± 95% confidence intervals shown. Green: upper location, purple: lower location. Large filled dots: group mean values ± S.E.M. Middle panel: comparison of median RT of each mouse between the 50–50 (open dots) and 90u-10 (filled dots) conditions. Right panel: distributions of change in median RT (90u-10 condition minus 50–50 condition) derived from middle panel. Upper location: median change = −20 ms, *p* = 0.031; Lower location: median change = −50 ms, *p* = 0.002. Red arrow: median; asterisk: *p* < 0.05; two-sided signed-rank and HB tests (see Methods). **b**, **c** Drift-diffusion modeling of RT distributions from spatial probability task; data from each mouse were fitted to the 4-parameter model, and the best-fit values of the parameters were obtained for each mouse (see Methods). Shown here are effects on two of the four parameters, conventions as in **a**: **b** rates of sensory evidence accumulation (drift rates). Upper location: median change = 0.06 a.u., *p* = 0.035; Lower location: median change = −0.26 a.u., *p* = 0.017. **c** Starting point. Upper location: median change = 0.01 a.u., *p* = 0.554; Lower location: median change = 0.04, *p* = 0.017. N.S. not statistically significant. See also Supplementary Fig. 2. Source data are provided as a Source Data file.

We next investigated the effect of spatial probability on the RTs of mice in this task. As a first step, we compared the median RTs of mice in the 90u-10 block versus the 50–50 block at each target location. We found that at the upper location, animals exhibited faster RTs during 90u-10 blocks versus 50–50 blocks (Fig. 2a, green data; median change = −20 ms, *p* = 0.031, signed-rank and HB tests), consistent with a potential higher expectation of target occurrence. Surprisingly, we found faster RTs at the lower location as well (Fig. 2a, purple data; median change = −50 ms, *p* = 0.002, signed-rank and HB tests).

To gain insight into the observation of faster median RTs at both target locations, we fitted the RT distributions from correct trials vs. incorrect trials (at each target location and for each mouse) with a two-choice drift-diffusion model (see Methods; Supplementary Fig. 2A–C). This approach allowed us to estimate in each block type and at each target location, the 4 model parameters for each mouse.

We found that when the target was at the upper location, the drift rate was higher in 90u-10 blocks relative to 50–50 blocks (Fig. 2b, green data, median change = +0.06 a.u., *p* = 0.035, signed-rank and HB tests), indicating a higher rate of evidence accumulation. None of the other three parameters were significantly different at the upper location between the two block types (Fig. 2c; Supplementary Fig. 2D, E; green data). By contrast, when the target was at the lower location, the drift rate was lower in 90u-10 blocks relative to 50–50 blocks (Fig. 2b, purple data, median change = −0.26 a.u., *p* = 0.017, signed-rank and HB tests), indicating a lower rate of evidence accumulation. In addition, the starting point was higher, i.e., biased towards the correct response (Fig. 2c, purple data, median change = +0.04 a.u., *p* = 0.017, signed-rank and HB tests), and the separation between decision boundaries exhibited a trend toward smaller values (Supplementary Fig. 2D, purple data, median change = −0.06 a.u., *p* = 0.039, not significant per signed-rank and HB tests). These observations indicated that, in the 90u-10 blocks, the threshold of evidence needed to trigger correct responses when the target was at the lower location was significantly reduced, or in other words, that mice sacrificed accuracy in favor of speed (Supplementary Fig. 2F; purple data).

These results revealed two key insights. First, that in the 90u-10 blocks, the rates of evidence accumulation at the two target locations were affected in opposite ways: higher drift rates at the upper location, and lower drift rates at the lower location are consistent with an increase and decrease in spatial expectation, respectively. Second, that the speeding up of RTs observed at both the upper and lower locations in the 90u-10 blocks were driven by different factors: at the upper location, faster RTs were consistent with the higher drift rates. By contrast, at the lower location, faster RTs were due to the reduced thresholds for triggering correct responses. If true, this lower threshold, i.e., the less stringent requirement for sensory evidence, would predict more errors when the target was at the lower location, a prediction that matched our observations (Fig. 1d, purple data).

Taken together*,* our results demonstrated space-specific effects of spatial probability on behavioral performance: compared with the 50–50 blocks, the 90u-10 blocks showed (1) increased accuracy, (2) improved perceptual sensitivity, and (3) faster evidence accumulation at the upper location, i.e., the target location with higher spatial probability; and a concurrent decrease in all three metrics at the lower location.

The behavioral results observed here were best explained by endogenous expectation-driven shifts of spatial attention rather than by any of the seven possible alternatives described next. (1) The space-specific nature of the results ruled out general arousal as a potential explanation for them. (2) Because the stimuli used in both block types were identical, and there were neither systematic differences in stimulus composition between block types at either target location (Supplementary Fig. 1M) nor were any external cues provided to the animals, the results could not be explained by exogenous accounts. Indeed, we note that any knowledge relating to the probabilities of target occurrence did not (and could not) provide any information about target orientation. (3) The effects could not be explained by differences in left–right responding: because our task design required operant responses at locations distinct from the target locations (putative loci of attention), biased responding either to the left or to the right would not yield any benefits for obtaining reward. Indeed, we found no differences in the left–right responding of the animals between the block types (Supplementary Fig. 1N). (4) They could not be accounted for by sequential effects such as feature priming^28^: when the target appeared at the same location in successive trials, say the upper location, there was no difference between the probabilities that the two targets were the same versus that they were different (both 50%) nor any difference in these probabilities between the 90u-10 and 50–50 conditions. In addition, when the same target appeared at a particular location in two successive trials, say the upper location, the probability of a correct response on the second trial was no different between the 90u-10 and 50–50 conditions (Supplementary Fig. 1Q). (5) The combination of long intertrial interval (median = 15.3 s with 95% CI of [13.8 s, 16.9 s] in the 50–50 condition, and median = 16.4 s with 95% CI of [14.9 s, 17.9 s] in the 90u-10 condition) with short stimulus duration (2 s) ruled out low-level accounts such as visual after effect^29^. (6) During these long intertrial intervals (median > 15 s in both block types), because mice needed to travel to the reward port, consume reward, and return to the touchscreen to initiate the next trial, the observed behavioral results could not be explained by mnemonic strategies, such as postural mediation^30^, to mark the location of higher probability. Instead, they had to rely on working memory.

Finally, (7) we tested whether the results could be accounted for by mice using motor strategies involving a systematic alteration of the head position in the 90u-10 condition. To this end, we used an automated, video-based head-tracking method (see Methods; Supplementary Fig. 3) to mark the head (snout-tip) position, as well as head direction (yaw/pitch angle) of mice in 3D during each trial in a subset of the animals (7/17). The results showed that the heads of mice moved along nearly identical trajectories in space in the 90u-10 and 50–50 conditions (Supplementary Fig. 4).

Consequently, the space-specific effects of spatial probability observed in the 90u-10 blocks are best explained by the mice inferring and holding information about the spatial probabilities in working memory within the 90u-10 sessions, in other words, by the action of an endogenous influence^31,32^.

### Exogenous capture of visuospatial-selective attention

**Fig. 3:**
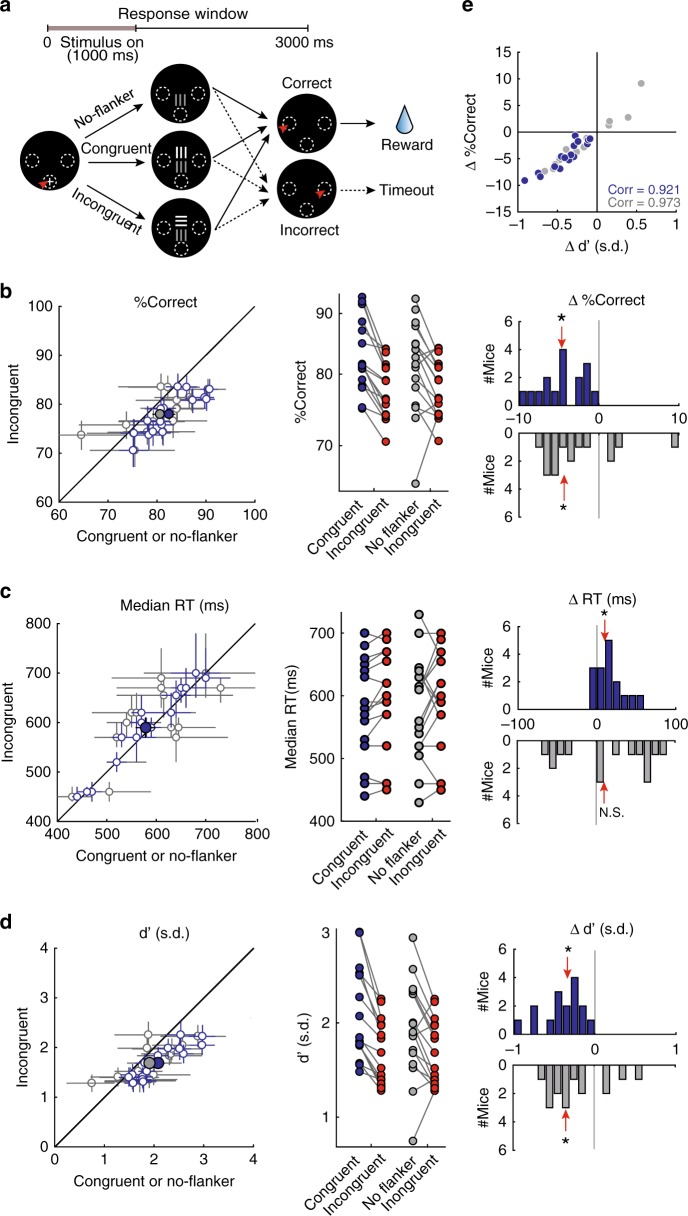
Incongruent flanker reduces accuracy and d’, and increases RTs, in freely behaving mice (*n* = 16 mice). **a** Schematic of task design. Upper row: trial timeline. Lower row: Screenshots of the display at different stages; shown from the mouse’s perspective. Following trial initiation, one or two grating stimuli were presented simultaneously. The target (one that yielded reward) was always presented at the lower location, and mice were trained to report the target’s orientation with an appropriate nose-touch; target orientation was either horizontal or vertical; only the vertical target is shown for simplicity. A second stimulus (flanker), when present, was behaviorally irrelevant, and was always presented at the upper location. Orientation of the flanker was either congruent (same) or incongruent (orthogonal) to the target across trials. Flanker contrast was varied parametrically in a randomly interleaved fashion across trials. For this figure, the data were analyzed by first collapsing across contrasts within each flanker condition. **b** Effect of incongruent flanker on response accuracy. Left panel: scatter plot comparing response accuracy to target’s orientation in incongruent trials (red data) versus either the congruent trials (blue data), or versus the no-flanker trials (gray data). Each open dot represents one mouse: mean ± 95% confidence intervals shown. Large filled dots: group mean ± S.E.M. Middle panel: Comparison of mean response accuracy (% correct) of each mouse between flanker conditions. Right panel: Distributions of changes in response accuracy; incongruent minus congruent (blue data, median change = −4.7%, *p* < 0.001), or incongruent minus no-flanker (gray data, median change = 4.4%, *p* = 0.026) trials. Red arrow: median; asterisk: *p* < 0.05, two-sided signed-rank and HB test. **c**, **d** Effect of incongruent flanker on median reaction time (**c**; blue data, median change = 10 ms, *p* = 0.019; gray data, median change = 10 ms, *p* = 0.385) and perceptual sensitivity (**d**; blue data, median change = −0.34 s.d., *p* < 0.001; gray data, median change = −0.35 s.d., *p* = 0.023); conventions as in **b**. **e** Plot of changes in response accuracy against changes in perceptual sensitivity. Blue data: incongruent trials versus congruent trials (Pearson’s correlation = 0.921, *p* < 0.001), gray data: incongruent trials versus no-flanker trials (Pearson’s correlation = 0.973, *p* < 0.001). See also Supplementary Fig. 5 and Supplementary Movie 2. Source data are provided as a Source Data file.

To study exogenous (bottom-up) control of visuospatial attention in freely behaving mice, we trained a majority of the same animals on a touchscreen version of the attentional flanker task used in humans^33,34^ (Fig. 3a; see Methods). Here, immediately upon trial initiation, up to two oriented gratings were presented on the screen at two locations along the vertical axis. The target grating, i.e., the one that yielded reward, was present on every trial, and always occurred at the lower location. The second, flanker grating, when present, always occurred at the upper location. Mice were rewarded for reporting correctly the orientation of the target grating while ignoring that of the flanker (when also present).

Trials were of three types: (a) singleton trials, in which only the target grating was presented, (b) incongruent flanker trials, in which the flanker grating was also presented simultaneously, with the orientation of flanker being orthogonal to that of the target, and (c) congruent flanker trials, in which the orientation of the flanker was identical to that of the target. In both the incongruent and congruent flanker trial types, the physical salience of the flanker, here, its visual contrast, was varied across trials from being less than to being greater than that the contrast of the target. This permitted the parametric investigation of the effect of the flanker on performance. All three trial types, as well as the different contrasts of the flankers, were interleaved randomly in each behavioral session.

Studies in humans^33,34^ show that incongruent flankers capture attention, and result in a greater number of error trials. Informed by this, and the observed asymmetry in mouse perceptual behavior between the upper versus lower locations (Supplementary Fig. 1H), we chose the lower location—the one with better perceptual performance—as the fixed location for the target stimulus, and the upper location as the fixed location for the flanker stimulus. This allowed us to test if a flanker affected behavioral performance in mice without a potential confound due to floor effects in performance.

### Effect of flanker on response accuracy and RTs

As a first step in analyzing the results from mice trained on this task (*n* = 16), we compared behavioral performance between incongruent and congruent flanker trials (Fig. 3b–d), and did so by collapsing data across flanker contrasts (Supplementary Fig. 5A; see Methods). We found that mice exhibited a significant impairment in response accuracy in the incongruent trials compared with the congruent trials (Fig. 3b, blue data; median change = −4.7%, *p* < 0.001, signed-rank and HB tests), and a significant increase in the median RTs (Fig. 3c, blue data; median change = +10 ms, *p* = 0.019, signed-rank and HB tests).

Upon partitioning response accuracy into perceptual sensitivity and decision criterion, we found a significant reduction in perceptual sensitivity in incongruent trials compared to the congruent trials (Fig. 3d, blue data; median change = −0.34 s.d., *p* < 0.001, signed-rank and HB tests), and no change in decision criterion (Supplementary Fig. 5B, blue data, *p* = 1, signed-rank and HB tests). The observed reduction in response accuracy was correlated strongly with the reduction in perceptual sensitivity across animals (Pearson’s correlation coefficient = 0.921, *p* < 0.001; Fig. 3e, blue data), and weakly with increases in the absolute value of criterion (Pearson’s correlation coefficient = −0.575, *p* = 0.02; Supplementary Fig. 5C, blue data).

Similar results were found upon comparing performance in the incongruent trials versus singleton (no-flanker) trials (Fig. 3b–d, gray data). These results showed that in mice, as in humans^33,34^, the incongruent flanker produced a reduction in accuracy and sensitivity, and a slowing down of reaction times, consistent with attention being captured by the flanker.

### Effect of flanker contrast on response accuracy

**Fig. 4:**
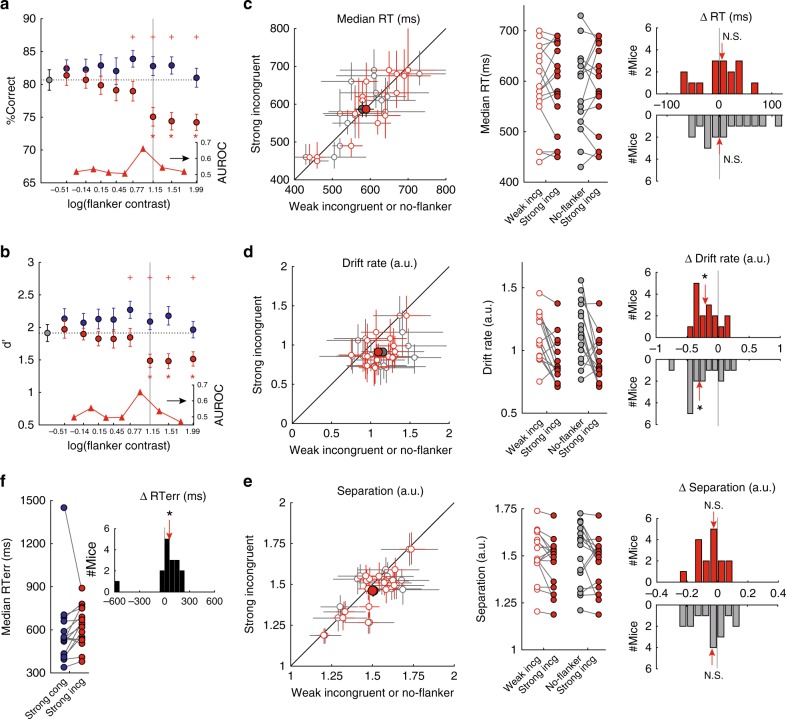
Incongruent flanker causes abrupt decrease in accuracy and d’, and reduction in rate of evidence accumulation, when stronger than target (*n* = 16 mice). **a** Plots of response accuracy as a function of flanker contrast (two-way ANOVA, effect of congruency, *p* < 0.001; effect of contrast, *p* = 0.021; interaction, *p* = 0.072). Gray vertical line: fixed contrast of the target. Gray data: no flanker; blue: congruent flanker; red: incongruent flanker. Data presented as group mean ± S.E.M. Red cross: *p* < 0.05; incongruent trials (red) were significantly different from congruent trials (blue) of that contrast level; red asterisk: *p* < 0.05; incongruent (red) trials were significantly different from no-flanker trials. Insets below (*y*-axis on right side): Ideal observer analysis quantifying area under the receiver operating characteristic (AUROC) between performance at successive pairs of contrasts in the incongruent condition, plotted at midpoint of each contrast pair. An abrupt increase in AUROC (sharp peak) was observed at flanker contrast close to that of target. Going forward, flanker contrast values 1–5 are referred to as ‘weak’ contrasts, and values 6–8, as “strong” contrasts. **b** Plots of perceptual sensitivity as a function of flanker contrast (two-way ANOVA, effect of congruency, *p* < 0.001; effect of contrast, *p* = 0.109; interaction, *p* = 0.393). Conventions as in **a**. **c**–**e** Effect of strong incongruent flanker on the median RTs (**c**), drift rate (**d**) and boundary separation (**e**), when compared with trials with weak incongruent flankers (red data), and trials with no flanker (gray data). Left panel: scatter plots. Each open circle represents individual mouse: mean ± 95% confidence intervals shown. Filled circles represent group mean ± S.E.M. Middle panel: Comparison of value for each mouse between flanker conditions. Right panel: distributions of the changes. Strong incongruent minus weak incongruent (red), or strong incongruent minus no-flanker (gray) trials. Red arrow: median of the distribution; asterisk: *p* < 0.05, N.S. not statistically significant, two-sided signed-rank and HB tests. **c** Red data: *p* = 0.698; gray data: *p* = 0.698. **d** Red data: *p* = 0.005; gray data: *p* = 0.006. **e** Red data: *p* = 0.049; gray data: *p* = 0.088. **f** Median RT in error trials: comparison of strong incongruent flanker trials versus strong congruent flanker trials. Left panel: comparison for each mouse. Right panel: distribution of change in median RT (*p* = 0.03, two-sided signed-rank tests). See also Supplementary Fig. 5. Source data are provided as a Source Data file.

Next, we analyzed the dependence of behavioral performance on the contrast of the incongruent flanker. We found that response accuracy in incongruent trials varied with flanker contrast in a striking manner (Fig. 4a, red data). When the contrast of the flanker was weak (relative to the target), response accuracy was neither significantly different from that in no-flanker trials (Fig. 4a, gray data) nor from that in congruent trials (Fig. 4a, blue data). However, when the contrast of the incongruent flanker just equaled that of the target, accuracy dropped significantly (Fig. 4a, red asterisk, *p* < 0.05 compared with no-flanker trials; red plus, *p* < 0.05 compared with congruent flanker trials. Two-way ANOVA followed by post-hoc comparisons with HB correction, main effect of congruency, *p* < 0.001; main effect of contrast, *p* = 0.021; congruency x contrast interaction, *p* = 0.072).

To quantify the abruptness of this transition in performance, we employed an ideal observer analysis and computed how well responses to successive contrasts of the incongruent flanker could be discriminated (Fig. 4a, inset at bottom). This analysis revealed a large difference in the discriminability of responses to successive values of contrast, precisely when the contrast of the incongruent flanker equaled that of the target (Fig. 4a, gray vertical line).

Upon partitioning accuracy into sensitivity and criterion, we found a similar abrupt change in perceptual sensitivity as a function of flanker contrast in incongruent trials (Fig. 4b, red data and Fig. 4b, inset at bottom). There was no effect of flanker contrast on the decision criterion (Supplementary Fig. 5D).

These results revealed that within the incongruent flanker trials, response accuracy and perceptual sensitivity were largely unaffected when flanker contrast was weaker than that of the target (weak contrasts), but decreased abruptly when the flanker contrast just surpassed that of the target (strong contrasts). In other words, the incongruent flanker was an effective distracter and disrupted performance only when it was as salient as, or more so than, the target.

### Effect of flanker contrast on RTs

We next tested for RT differences between strong flanker trials (contrast values of #6–#8) versus weak flanker trials (with contrast values #1–#5) when the flanker was incongruent. Surprisingly, we found no difference in the median RTs (Fig. 4c, *p* = 0.991, signed-rank test).

To investigate this result in greater detail, we tested if there were any differences in the distributions of RTs (as opposed to just the median values), by applying the drift-diffusion modeling approach (Supplementary Fig. 5E; see Methods).

We found that the drift rates were significantly lower in the strong incongruent flanker condition (Fig. 4d, red data, median change = −0.207 a.u., *p* = 0.005, signed-rank and HB tests), and the boundary separation showed a trend towards being smaller (Fig. 4e, red data; median change = −0.025 a.u., *p* = 0.049, signed-rank and HB tests; Fig. 4e is not significant). There were no systematic differences in the other parameters (Supplementary Fig. 5F, G, red data; starting point, *p* = 0.642; non-decisional constant, *p* = 0.501, signed-rank tests). Thus, the overall absence of change in the RT (Fig. 4c) was the result of two competing effects: a reduction in the drift rate (consistent with distraction by the strong incongruent flanker), and a trend towards reduction in the threshold to correct responses (as in the spatial probability task).

The above findings also held true when the strong incongruent flanker trials were compared with the no flanker trials (Fig. 4c–e and Supplementary Fig. 5F, G; gray data), and, as expected, also when compared with the strong congruent flanker trials (Supplementary Fig. 5H–L).

### Effect of flanker on error trials

As a final step in the analysis, we examined just the error trials when the flanker contrast was strong, specifically comparing the incongruent versus congruent conditions. The rationale was as follows. In the strong congruent flanker condition, because the flanker contains the same orientation information as the target, any attentional disruption by the strong flanker is not expected to produce systematic errors. In other words, the error trials in this condition reflect errors due to nonspecific factors such as limits in learning the discrimination, failure in reporting it, and/or attending elsewhere altogether, rather than attention capture (distraction) by the flanker. By contrast, the error trials in the strong incongruent flanker condition reflect errors due both to distraction by the flanker, as well as due to these nonspecific factors. Therefore, a direct comparison of these two sets of error trials can provide additional insight, specifically, into the processes underlying attentional capture. We found that the median RTs were longer in error trials with strong incongruent flankers, compared to error trials with strong congruent flankers (Fig. 4f, median change = +57.5 ms, *p* = 0.03, signed-rank test).

Taken together, mouse behavior in the flanker task exhibits hallmarks of attention being captured by an exogenous or bottom-up distracter (the incongruent flanker). In addition, parametric variation of the contrast of the incongruent flanker revealed an abrupt increase in the efficacy of the flanker to serve as a distracter when its salience just exceeded that of the target.

## Discussion

In this study, we demonstrated that freely moving mice exhibit classic behavioral signatures of visuospatial selective attention under both endogenous as well as exogenous control. This is the first approach, to the best of our knowledge, which accomplishes three critical goals: (a) the demonstration of primate-like visuospatial selective attention, (b) in freely behaving animals, and (c) in a species that facilitates the use of diverse genetically based tools for neural interrogation (Supplementary Fig. 1R). The hallmark of “primate-like” visuospatial-selective attention is spatially-specific changes (improvements and decrements) in visual perceptual performance^26,35,36^, demonstrated while ensuring that the effects of attention can be disambiguated from potential sensory or motor effects. Most previous studies in non-primate species achieve some, but not all, of these features. This includes work using the n-choice serial reaction time (n-CSRT) task^37^ and its variants in (freely behaving) rodents^38–42^, as well as work in pigeons^43–45^. The two exceptions are a recent study in chickens^5^ (a species not ideal for the use of modern genetically based tools for neural interrogation), and a recent study in mice^6^ (which was performed in a head-fixed preparation). The tasks developed here, therefore, serve as an excellent substrate for future investigations into the cell-type specific neural signatures as well as the neural control of visuospatial selective attention in freely behaving mice.

Our tasks involved presenting stimuli at two possible locations along the vertical axis: one in the upper periphery and the other closer to the center. Orthogonalizing the axes of stimulus locations and nose-touch locations allowed us to unambiguously attribute any observed changes in behavior to motor vs. sensory or cognitive sources. As a by-product of this design, we found asymmetry in mouse visual performance between the upper and lower locations, similar to that reported in humans^46,47^. In addition, our tasks were designed as two alternative forced choice discriminations, as opposed to go/no-go detection tasks, thereby minimizing overall response bias^48^, and notably, allowing the quantification of RTs for trials corresponding to both choices (i.e., trials with vertical as well as horizontal targets, in our case). The orientation difference that the mice had to discriminate in our tasks was maintained fixed at the maximal value of 90°. Nonetheless, the tasks can be extended in the future by using smaller orientation differences^6,49^ or by testing a range of orientation differences.

The approach of manipulating spatial probability to affect endogenous control of spatial attention was inspired by human studies that have shown that probability manipulation can itself serve as a spatial attentional cue^22,23^. Indeed, direct comparison of the results between trials with an explicit spatial cue versus with a probability manipulation revealed no differences from a Bayesian observer’s perspective^24^. Consequently, in the 90u-10 blocks, trials with the target at the upper location may be treated as trials with a “valid” cue (90% of trials), whereas trials with the target at the lower location may be treated as trials with an “invalid” cue (10% of trials). In primate studies, validly cued trials represent the “attend towards” condition, and the invalidly cued trials, the “attend away” condition, and the difference in performance between these two conditions is used to characterize the effect of spatial attention. We performed this comparison as well, by computing the differences in performance between the upper and lower locations in the 90u-10 blocks (Supplementary Fig. 1O, P; maroon data), relative to those in baseline (the 50–50 block; teal data). We found an improvement of 6.5% in accuracy (difference between the medians) and 0.57 s.d. in d’. In other words, these are the net improvements in the “attend towards” condition compared with the “attend away” condition. This observed improvement in accuracy is within the range (5–15%) of previous reports across a variety of primate attention tasks^46,50–53^. Using net improvement in accuracy as the metric, we also examined the significance of behavioral results for each individual mouse. We found that among the 17 mice tested, five exhibited significant improvement (*p* < 0.05, permutation test) in response accuracy and perceptual sensitivity (d’). That mice were able to infer spatial probabilities^54^ and shift attention appropriately even in the absence of an explicit spatial cue, and in a freely behaving condition, is significant.

In these experiments, we measured head positions in 3D, but did not measure eye-in-orbit positions. However, lack of knowledge of eye positions did not present a confound for the unambiguous interpretation of our behavioral results in the context of visuospatial selective attention. Eye movements that could improve performance in the 90u-10 condition would involve a systematic direction of eye gaze toward the upper location, which, in turn would serve as explicit evidence for the overt, as opposed to covert, direction of spatial attention: It is well-established that shifts in gaze direction produce yoked shifts in selective attention to the target of gaze^55–58^; indeed, in the natural world, primates and other vertebrates (birds, cats, rodents, bats, etc.) routinely attend selectively to spatial locations by making orienting movements of the eyes, ears, or head toward those locations. Here, because our goal was to demonstrate signatures of attention, rather than the demonstration, specifically, of overt vs. covert versions of it, measurement of eye positions was not necessary, consistent with past work in humans and animals^6,23^. This is also consistent with the fact that rodents use head and body movements predominantly (rather than eye movements) to orient to targets; their lack of a fovea likely makes goal-directed eye movements less useful for accessing perceptual details^59,60^.

The results from our touchscreen-based flanker task are consistent with results from the classic flanker task of attention in humans^33,34^ including contrast-dependent effects^61,62^. Although the task demonstrates exogenous disruption of attention, it requires mice to pay attention to the fixed location of the (reward-yielding) target, resulting in attention being directed to the target in an endogenously driven manner as well. Thus, each trial of this task involves constant competition between purely exogenous influences due to the flanker stimulus, and endogenous plus exogenous influences towards the target. It is only when the flanker becomes sufficiently salient that it is able to overcome the endogenously highlighted target to capture attention (Fig. 4a, b).

One may argue that instead of viewing the stimuli as target versus flanker, mice may view the two gratings together as one larger composite object, and perform feature discrimination on this composite. We can rule out this alternative for two reasons. First, it fails to explain the differential performance between the congruent and incongruent trials. Specifically, since all mice encounter four different orientation patterns of the composite (target + flanker) stimulus, namely, upper orientation: lower orientation = vertical (V):horizontal (H), H:V, V:V, and H:H, there is no a priori reason why mice would perform substantially better for two patterns out of the four (V:V, and H:H, i.e., congruent trials) than the other two patterns (V:H, and H:V, i.e., incongruent trials). Second, it also fails to explain the contrast dependence of performance on incongruent trials. This is best illustrated by comparing the incongruent trials on which the target and flanker were of equal contrast (Fig. 4a, red data, 6th datapoint from left, “equal flanker data”), with the incongruent trials on which the flanker was of weaker contrast than the target (Fig. 4a, red data, the first five datapoints, “weak flanker data”). The equal flanker data indicate that mice learn to associate the incongruent composite patterns of H:V to left nose-touch and V:H to right nose-touch to an average level of ~75%. The composite stimulus patterns corresponding to the weak flanker data involve dimmer upper portions of the composite stimulus—as though obtained by applying a semitransparent mask or neutral density filter onto it. The poorer visibility of the upper portions of these composite stimuli ought to make discrimination of the composite more difficult, predicting a reduction in performance. In direct contrast to this prediction, mice perform significantly better to these “less visible” composite stimulus patterns in which the upper portion became dimmer (i.e., when the flanker became weaker). Therefore, our data suggest that mice resolved the target and the flanker as separate gratings, and responded selectively based on the target orientation when the flanker was not salient.

The use of the mouse model has started yielding insights into neural circuits related broadly to visual detection/discrimination as well as sensory selection and attention. Sustained attention has been shown to rely on prefrontal parvalbumin interneurons in mice^63^. Activation of the cingulate cortex has been shown to sharpen the orientation tuning of V1 neurons and thereby modulates orientation discrimination behavior in mice, mimicking the effects of attention^64,65^. Likewise, activation of striatal neurons has been shown to bias perceptual choices of mice in a visual detection task^66^. In addition, the ability to selectively attend to a stimulus of one sensory modality versus another has been shown to depend on a thalamic circuit^10,11^. These findings, which involve optogenetic manipulation of specific groups of neurons, or calcium imaging with genetically encoded calcium sensors, highlight the power of the mouse model for precise targeting of neurons in a cell-type specific manner.

Nonetheless, there is still a range of questions related to visuospatial selective attention that remain unanswered. The tasks developed in this study offer key advantages to investigate them. First, the combination of the use of small, spatially restricted stimuli, and the dissociation of locus of attention from locus of action, afford greater clarity in interpreting neural correlates as well as circuit mechanisms of selective spatial attention. Furthermore, our parameterized task design (of the flanker task) is well-suited to reveal whether neural signatures of target selection and distracter suppression in the oculomotor pathway are explicitly categorical, as hypothesized recently^67,68^. Second, our two tasks also demonstrate separately the effects of endogenous and exogenous control of attention respectively, making them well-suited for investigating the underlying circuit mechanisms^68–70^. This is advantageous because in some spatial cueing paradigms, the use of a spatial cue can result in asymmetric sensory stimulation, with the cued location receiving greater magnitude of input (especially when the cue precedes the stimulus with little to no delay), thereby making it difficult to disentangle the contribution of exogenous versus endogenous influences. Thirdly, behavioral states of animals have been shown to have a significant effect on neural representations^17–19^. The ability to characterize complex behavior in the unrestrained state, therefore, affords the power to obtain a richer picture of its neural underpinnings. This is especially true for visuospatial selective attention, a function that animals employ heavily when interacting freely with their environments, and for rodents that orient to targets predominantly with head and body movements (rather than eye movements)^59,60^. Here, our demonstration that freely behaving mice exhibit systematic changes in behavioral performance due to selective spatial attention, opens the door for insights into potential behavioral state-dependent differences in the neural basis of this important cognitive function.

## Methods

### Animals

All mice were C57Bl6/J strain (male adults; >12 weeks old) and were purchased from the Jackson Laboratory. Upon arrival, mice were housed in a colony where temperature (~75 F) and humidity (~55%) were controlled on a 12:12 h light:dark cycle. At least 1 week of acclimation period was allowed with food and water ad libitum before water restriction was initiated. Experiments were all carried out in the light phase. All procedures followed the NIH guidelines and were approved by the Johns Hopkins University Animal Care and Use Committee.

### Water restriction

Mice were water-restricted following protocols described by Guo et al.^71^ with a few modifications. Briefly, mice were individually housed, and administered 1 mL water per day to taper their body weight down to 80–85% of each animal’s baseline, over the course of 5–7 days. During behavioral training/testing, mice received 10 µL of water for every correct response.

### Apparatus

Behavioral training and testing were performed in soundproof operant chambers equipped with a touchscreen (K-Limbic, and Med-Associates Inc.), a reward port (fluid well), infrared video cameras, a house light, and a magazine light above the reward port. The reward port was located at the opposite wall of the chamber relative to the touchscreen (Fig. 1a). Two custom modifications were introduced that limited the area of the touchscreen available for exploration by the freely behaving mice, thereby minimizing false-alarm triggers due to accidental touches. First, mice were placed within a clear plexiglass tube that ran from the touchscreen to the reward port. The diameter of the tube (5 cm) was large enough to allow mice to run back and forth from the touchscreen to the reward port, to groom and to behave naturally. Second, a thin plexiglass mask (3 mm thickness) was placed 3 mm in front of the touchscreen with three holes corresponding to the locations at which the mouse was allowed interact with the screen by a nose-touch (Fig. 1a; Supplementary Fig. 1A). The holes, each 1 cm in diameter, were drilled in the mask in an inverted triangle configuration: “left” and “right” holes were placed 3 cm apart (center-to-center) along the base of the triangle, and a “central” hole, at the apex of the triangle, was 1.5 cm below the midpoint of the base (Fig. 1a). All experimental procedures were executed using control software (K-limbic v1.30, Med-Associates).

### Visual stimuli

Visual stimuli (bright objects on a dark background; background luminance = 1.32 cd/m^2^) were generated using MATLAB (Mathworks) and imported into the K-Limbic system as jpeg images. All stimuli were small, of size 60 pixels × 60 pixels, i.e., 12 mm × 12 mm, and subtended a visual angle of 25° at a viewing distance of 20 mm from the mask (Supplementary Fig. 1A). A zeroing cross (luminance = 130 cd/m^2^) was presented in the central hole and had to be touched to initiate each trial. The experimental stimuli were oriented gratings (horizontal or vertical orientation), generated using a square wave of spatial frequency 24 pixels/cycle (0.1 cycles per degree). This frequency was chosen because it lies within the range of spatial frequencies shown to be effective for visual discrimination in mice^9^). The dark phase of the cycle was black (luminance, L_dark_ = 1.32 cd/m^2^; same as the background), and the luminance of the bright phase (L_bright_) was varied between 1.73 cd/m^2^ and 130 cd/m^2^ to control its contrast (flanker task, see below). Contrast was defined as the ratio of difference in luminance between the bright and dark phases of the grating over that of the dark phase: contrast = (L_bright_ – L_dark_)/L_dark_ (units of fractional change).

### Experimental procedure and behavioral training

Each mouse was run for one 30-min behavioral session per day, with each session yielding 80–180 trials. Each behavioral session began with a 10 s acclimation period, during which mice were allowed to explore the environment with the lights on and to retrieve a bolus (10 µL) of free water at the reward port. Following this, lights shut off and the zeroing cross to start the first trial appeared on the screen. The cross flashed once every 10 s until touched, and the flash was accompanied by a short beep of 600 Hz for 30 ms, to induce the mouse to approach and begin the trial. Upon trial initiation, the cross vanished, and the visual stimulus (or stimuli) were immediately presented for a duration of 1–3 s depending on the task (see below).

Mice were trained to report the information contained in the target grating, namely, its orientation, by nose-touching within the correct response hole (vertical target grating → nose-touch in left response hole; horizontal target grating → nose-touch in right response hole). A correct response triggered a tone (600 Hz, 1 sec), the turning on of the magazine light above the reward port, and the delivery of 10 µL of water at the reward port. Mice turned away from the screen, ran to the liquid well, consumed the reward, and ran back to face the touchscreen in order to begin the next trial. Mouse head entry into the reward port was detected by an infrared sensor which caused the magazine light to turn off, and the zeroing cross (for the next trial) to be presented on the touchscreen. An incorrect response triggered the turning on of both the house light and the magazine light for 5 s as a punishment/timeout; the next trial could not be initiated until the end of timeout. A failure to respond within 3 s of stimulus presentation resulted in the stimulus vanishing and the zeroing cross being presented immediately (without a timeout penalty) for initiation of the next trial. Well-trained animals failed to respond on fewer than 5% of the total number of trials, and there were no systematic differences in the proportion of such missed trials between different conditions.

Within each daily 30-min behavioral session, mice consumed ~1 mL of water. If a mouse failed to collect enough water from the behavioral session, they were provided with a water supplement using a small plastic dish in their home cage. The specific amount of supplement was customized depending on individual animal’s body weight, the training phase it was in, and the motivational drive observed during the experiment.

### Single-stimulus discrimination task

Upon trial initiation, a single, full contrast, grating stimulus (target, contrast = 97.5; size = 60 × 60 pixels^2^, 25°, 2.5 cycles) was presented above the central hole, aligned along the elevation with the left and right holes. The stimulus was presented for a duration of 3 s, and mice were required to report its orientation with the appropriate nose-touch (Supplementary Fig. 1A).

### Spatial probability task

Upon trial initiation, a single grating stimulus (target, 60 × 60 pixels^2^, 25°, 2.5 cycle, 2 s, contrast = 14.2) was presented at one of two possible locations: an upper location (the center of the grating was 90 pixels or 37.5° above the center of the central hole), and a lower location (30 pixels or 12.5° above the central hole). The stimulus was presented for a duration of 2 s, and mice were required to report its orientation with the appropriate nose-touch within 3 s (Fig. 1c). Trials were run in blocks of two kinds, with different probabilities of target occurrence at the upper (and lower) locations; blocks were interleaved pseudo-randomly across days. In the “50-50” blocks, the probability that the target would appear at the upper location on any trial was 50% (and at the lower location, also 50%). In the “90u-10” blocks, the probability, on any trial, that the target would appear at the upper location was 90% (and at the lower location, 10%). This design allowed us to test if spatial expectation altered behavioral performance. To train mice on this spatial probability task, they were first trained on the single stimulus discrimination task (with the target always presented midway between the lower and upper locations), following which the two possible target locations with corresponding spatial probabilities were introduced.

### Flanker task

Upon trial initiation, either one stimulus (target, 60 × 60 pixels^2^, 25°, 2.5 cycle, 1 s, contrast = 14.2) was presented at the lower location, or two stimuli were presented simultaneously, with the target at the lower location and a second flanker at the upper location. Flankers were of the same size and spatial frequency as the target, but with contrast at 8 different levels: 0.31, 0.73, 1.42, 2.79, 5.84, 14.2, 34.4, and 97.5. The orientation of the flanker was either identical to that of the target (congruent trial) or orthogonal to that of the target (incongruent trial). The stimulus (stimuli) was (were) presented for a duration of 1 s, and mice were required to report orientation of the target grating with the appropriate nose-touch within 3 s (Fig. 3a). All types of trials (no flanker, congruent, incongruent) and flanker contrasts were interleaved randomly within each daily session. To train mice on this flanker task, they were first trained on the single-stimulus discrimination task (with the target always at the lower location), following which, a flanker was introduced at the upper location with progressively increasing contrast over training days.

### Behavioral measurements

Response accuracy (% correct) was calculated as the number of correct trials divided by the total number of trials responded (correct plus incorrect). Reaction time (RT) was defined as the time between the start of stimulus presentation and response nose-touch, both detected by the touchscreen.

### Signal detection analysis (sensitivity and criterion)

In the framework of signal detection theory (Supplementary Fig. 1J), we assigned the correct vertical trials as “hits”, incorrect vertical trials as “misses”, correct horizontal trials as “correct rejections”, and incorrect horizontal trials as “false alarms”, and calculated the perceptual sensitivity (d’) and criterion (c) accordingly^35^. Because of the inherent symmetry in 2-AFC tasks, this calculation was independent of which grating orientation— vertical or horizontal—was assigned as “signal” and which as “noise”. In addition, in 2-AFC tasks, compared with c = 0 (the optimal or unbiased value), a positive value of c as well as a negative value of c of the same magnitude, both caused a similar reduction in accuracy (Supplementary Fig. 1J; upper row, right panels). The magnitude of c, i.e., the deviation of criterion from the unbiased value, therefore, captured its effects on overall accuracy better than its signed value. For this reason, we used the absolute value of c (| c |) as the relevant metric of decision criterion (Supplementary Figs. 1K, L and 5B–D). To compare criteria between two conditions, we computed the change in criterion as ∆c = | c_2_ | − | c_1_ | , where c_1_ and c_2_ were the criteria in the two conditions, respectively.

### Subject inclusion/exclusion

A total of 25 mice were used in this study. All 25 were trained on the single-stimulus discrimination task, and of these, *n* = 20 mice satisfied the inclusion criterion: % correct >70% (yielding the data shown in Supplementary Fig. 1B–G). All 25 mice were also trained on the spatial probability task, and of these, *n* = 17 mice satisfied the inclusion criterion for the spatial probability task: overall % correct in the (baseline) 50–50 condition >70% (data shown in Figs. 1, 2, and Supplementary Fig. 1I, K–Q). A total of 18 mice were trained on the flanker task, and of these, *n* = 16 mice satisfied the inclusion criterion for the flanker task: overall % correct across all flanker contrasts >70% (data shown in Figs. 3 and 4). Between experiments, mice were well rested for at least few weeks with food and water ad libitum, before the next water restriction was initiated.

### Trial inclusion/exclusion

Towards the end of a behavioral session, when mice had received a sizeable proportion of their daily water intake, they were observed to become less engaged in the task. This was reflected in their behavioral metrics: they tended to wait longer to initiate the next trial, and their performance deteriorated. To avoid confounds due to loss of motivation towards the end of sessions, we developed an unbiased procedure to identify and exclude such trials. To this end, we pooled data across all mice and all sessions, treating them as coming from one session of a single “mouse”. We then binned the data by trial number within the session, computed the discrimination performance in each bin (% correct), and plotted it as a function of trial number within session (Supplementary Figs. 1D, I, and 5A). In addition, we used a bootstrapping method to compute the 95% confidence interval for this value. As expected, we found that the performance became highly variable and dropped towards chance for trials towards the end of the session (Supplementary Figs. 1D, I, and 5A). Using this data, we developed the following exclusion criterion: Trials q and above were dropped if the qth trial was the first trial at which at least one of the following two conditions was satisfied: (a) the performance was statistically indistinguishable from chance on the qth trial and for the majority (3/5) of the next 5 trials (including the qth), (b) the number of observations in qth trial was below 25% of the maximum possible number of observations for each trial (mice*sessions), thereby signaling substantially reduced statistical power available to reliably compare performance to chance.

For the single-stimulus discrimination task, q was determined to be the 122nd trial using pooled data from all the sessions of the 20 mice (Supplementary Fig. 1D). Thus, trials 122 and above were dropped from each session. This resulted in the exclusion of 3.7% of the trials (229 out of 6176 trials).

For the spatial probability task, q was determined to be the 141st trial using pooled data from all the 50–50 sessions (irrespective of the stimulus location) of the 17 mice (Supplementary Fig. 1I). Thus, trials 141 and above were dropped from each 50–50 session of the spatial probability task. This resulted in the exclusion of 5.7% of the trials (781 out of 13772 trials). This same value of q was used to drop trials from the 90-10 sessions to ensure unbiased treatment of both conditions; this resulted in the exclusion of 4.10% of the trials (656 out of 15522 trials).

For the flanker task, q was determined to be the 131st trial using pooled data from all sessions (irrespective of the trial type and flanker contrast) of the 16 mice (Supplementary Fig. 5A). Thus, trials 131 and above were dropped from each session of the flanker task. This resulted in the exclusion of 2.53% of the trials (834 out of 32959 trials).

### Drift-diffusion modeling of RT distributions

To shed light on potential mechanisms underlying observed RT distributions, we applied the drift-diffusion model to our RT data^72,73^. This model hypothesizes that a subject (decision maker) collects information from the sensory stimulus via sequential sampling, causing sensory evidence to accrue for or against a particular option (usually binary) during the viewing of the stimulus. A decision is said to be made when the accumulating evidence reaches an (abstract) internal threshold of the subject. This process of evidence accumulation, together with the processes of sensory encoding and motor execution, as well as threshold crossing, are said to determine the RT observed on each trial.

We used a standard version of the model that consists of four independent variables (Supplementary Fig. 2A)^72,73^: (1) the drift rate, which represents how fast sensory evidence accrues; (2) the boundary separation, which represents the bar that subject sets for the decision to be made; (3) the starting point, which represents the (prior) biases the subject might have favoring one versus the other option (=0.5 when unbiased); and a (4) non-decisional constant, which accounts for the time spent in sensory encoding and motor execution. In the case of our tasks, there was no reason for the drift rate to be different between vertical versus horizontal gratings, and therefore, we merged both type of trials (trials with a horizontal target grating and trials with a vertical target grating). We treated “correct” response and “incorrect” response as the two binary options, and fit the diffusion model to the RT distributions of correct versus incorrect trials using the fast-DM-30 toolbox with the maximum likelihood option to gain estimates of those four parameters for each individual mouse^74^.

To obtain accurate estimates of model parameters, it is important to drop trials with outlier values of RTs (too fast or too slow trials), as per the established approach in drift-diffusion modeling^74^. We developed an unbiased procedure to identify and exclude inordinately fast or slow trials. We reasoned that on trials with RTs that are so short as to not allow mice sufficient time to accumulate sensory evidence, performance would be consistently poor because mice would be forced to guess. Similarly, on trials with RTs that are so long (far exceeding stimulus offset) as to extinguish the trace of sensory evidence from their short-term memory^75,76^, performance would be consistently poor because animals would be forced to guess. To apply this heuristic, we pooled RT data across all mice and all sessions, treating them as coming from one session of a single mouse. We then binned this RT distribution into 50 ms bins and for each bin, computed the response accuracy and the 95% confidence intervals (using a bootstrapping method). We then identified short and long RT bins for which the response accuracy was statistically indistinguishable from chance (Supplementary Figs. 2B and 5E).

For the spatial probability task, using pooled data from all 50–50 sessions of the 17 mice (irrespective of the stimulus location), we determined that trials with RTs shorter than 300 ms or longer than 2550 ms were outliers (Supplementary Fig. 2B). This resulted in the exclusion of 6.3% of the trials (815 out of 12991 trials). The same RT exclusion range was used for data from the 90u-10 sessions to ensure unbiased treatment of both conditions. This resulted in the exclusion of 9% of the trials (1335 out of 14866 trials). Outlier trial exclusion did not alter the effects of spatial probability on median RT in Fig. 2a (Supplementary Fig. 2C).

For the flanker task, using pooled data from all sessions of the 16 mice (irrespective of trial type and flanker contrast), we determined that trials with RTs shorter than 250 ms or longer than 1900 ms were outliers (Supplementary Fig. 5E). This resulted in the exclusion of 5.06% of the trials (1625 out of 32125 trials).

### Markerless automated video-based head-tracking

A custom software package (MAHTS) written in FIJI (ImageJ v1.52p)^77^ and MATLAB (R2018b) was developed to perform offline head-tracking of freely behaving mice from behavioral videos. Raw behavioral videos of the freely behaving mice were recorded from the top view and lateral view simultaneously during the spatial probability task (frame rate = 30 Hz). This was done in a subset of behavioral sessions for seven mice. From these raw videos, snippets corresponding to each trial were extracted in an automated fashion (custom code, MATLAB). The first frame of each snippet corresponded to trial initiation (nose-touch to the zeroing cross within the central port), and the last frame to trial completion (first nose-touch within one of the response ports). For each behavioral session of a mouse, video snippets across all the trials were concatenated to produce master videos—one for the top view and one for the lateral view. This resulted in a total of 42 master videos for each view (7 mice × 2 behavioral blocks (50–50 and 90u-10) × 3 sessions for each block).

The master videos were first preprocessed in FIJI (using a custom written plug-in). Preprocessing involved cropping the master videos to show just the head of the mouse (Supplementary Fig. 3A, B), smoothing each frame with a median filter, enhancing the contrast, subtracting background, and segmenting the image with a built-in, automated thresholding method.

These binarized (top view and lateral view) master videos from FIJI (Supplementary Fig. 3C, D) were then analyzed, frame-by-frame, in MATLAB (custom functions) to extract the position of the tip of the snout and the angle of orientation of the head in each frame. To this end, the enveloping “snout cone” of the mouse (Supplementary Fig. 3C, D; red dashed lines) was estimated in the top view and lateral view. This was done by fitting a best-fit straight line to the points lying on the edges of the binarized mouse head in each frame. The point of intersection of the two red lines, i.e., the vertex of the snout cone (Supplementary Fig. 3C, D; red circle), was taken as the estimate of the tip of the snout. Estimates from the top and lateral view together uniquely determined the position of the tip of the snout (or the relevant information about the position of the head) in 3D space at each instant.

The orientation of the head (head direction) in the top view (i.e., yaw angle, *θ*) was estimated as the angle of the line bisecting the snout cone (Supplementary Fig. 3A, C; red solid line) from the vertical. The orientation of the head in the lateral view (i.e., pitch angle, φ) was estimated as the angle of the top edge (Supplementary Fig. 3B, D; red solid line) from the horizontal. These estimates exploited the fact that the eyes of the mouse are positioned along the top edge in the lateral view, and centered on the midline of the snout in the top view, as a result of which these lines represented the projections of the head orientation axis in 3D space onto the top and lateral views.

To validate the automated method, we compared its estimates of head position and direction from a subset (1020) of the frames, with those obtained manually by two human researchers (WKY and RP). For each frame, the researchers manually marked the position of the snout cone (Supplementary Fig. 3E, F; white dashed lines—WKY, blue dashed lines—RP), thereby estimating the tip of the snout (white and blue squares, respectively) as well as the head-direction angles (white and blue solid lines, respectively). Comparison of these manual estimates from the automated versus manual methods revealed close correspondence (Supplementary Fig. 3G, H), thereby confirming the reliability of the automated method. (The automated method was necessary because it provided a >50-fold reduction, compared with the manual method, in the time taken to estimate head position and direction from videos.)

The extracted *x*, *y*, and *z* coordinates of head position were converted from pixels to mm based on the scaling factor (resolution) of the videos; the origin ([*x*, *y*, *z*] = [0, 0, 0]) was assigned to be the starting position (within the central hole) of each trial. Because trial-to-trial reaction times were variable, the number of frames per trial, and therefore, the number of values of *x*, *y*, or *z* positions per trial, was variable. These positions as well as the head-direction angles were plotted by RT quantile to allow averaging across sessions/animals and comparisons across conditions. (Supplementary Fig. 4C–G).

### Statistical analyses

All analyses and statistical tests were performed in MATLAB. For cross-condition comparisons (Figs. 1–4), for each behavioral metric (% correct, RT, etc.), the non-parametric Wilcoxon signed-rank test (two-tailed) was used to test if the median of the distribution of the change in the metric between conditions (e.g., 90u-10 vs. 50–50, or incongruent vs. congruent) was different from zero, followed by the Holm–Bonferroni test for multiple comparisons across stimulus locations (spatial probability task) or flanker conditions (flanker task). The application of this statistical procedure is referred to in an abbreviated fashion as “signed-rank and HB tests” in the text. For correlation analysis (Fig. 1f, g, 3e), the Pearson correlation coefficient and corresponding *p*-value were calculated for paired data using corrcoef command in the MATLAB. For flanker contrast-dependent analysis (Fig. 4a, b), two-way ANOVA was first used to examine the effect of flanker congruency, and the effect of flanker contrast. Post-hoc paired comparisons were then performed using Student’s *t* test in selected pairs, followed by the Holm–Bonferroni test (HB test) for multiple comparisons. Statistical significance was defined as *p* < 0.05. For statistical comparison of head trajectories (Supplementary Fig. 4C–G), to compare the trajectory of each head position/direction parameter between the two conditions (50–50 and 90u-10), we performed permutation tests (2000 shuffles) for the data for the left response location (solid lines) as well as the right response location (dashed lines). The permutation tests were performed on the difference between the mean trajectories. The resulting p-values from the permutation tests were corrected for multiple comparisons at the 0.05 level (8 comparisons = 4 comparisons for datasets for each of the two response locations). The samples that showed a significant difference following correction for multiple comparisons are indicated either as asterisk (*, data for left response location) or cross (+, data for right response location).

### Reporting summary

Further information on research design is available in the Nature Research Reporting Summary linked to this article.

## Supplementary information

Supplementary information

Description of Additional Supplementary Information

Supplementary Movie 1

Supplementary Movie 2

Reporting summary

## Data Availability

Data supporting the findings of this study are available at 10.6084/m9.figshare.11987637.v1.

## Code availability

The custom software package for head-tracking (MAHTS, written in MATLAB and FIJI) is available from the corresponding author upon reasonable request.
