# Supplementary material for: Endogenous and exogenous control of visuospatial selective attention in freely behaving mice
Source: Nat Commun. 2020 Apr 24;11:1986. doi: 10.1038/s41467-020-15909-2 (PMC7181831; doi:10.1038/s41467-020-15909-2)
Supplement: Supplementary file 1 — Supplementary information [file 41467_2020_15909_MOESM1_ESM.pdf]

Supplementary Information

Endogenous and exogenous control of visuospatial selective attention in freely behaving mice

Wen-Kai You and Shreesh P. Mysore

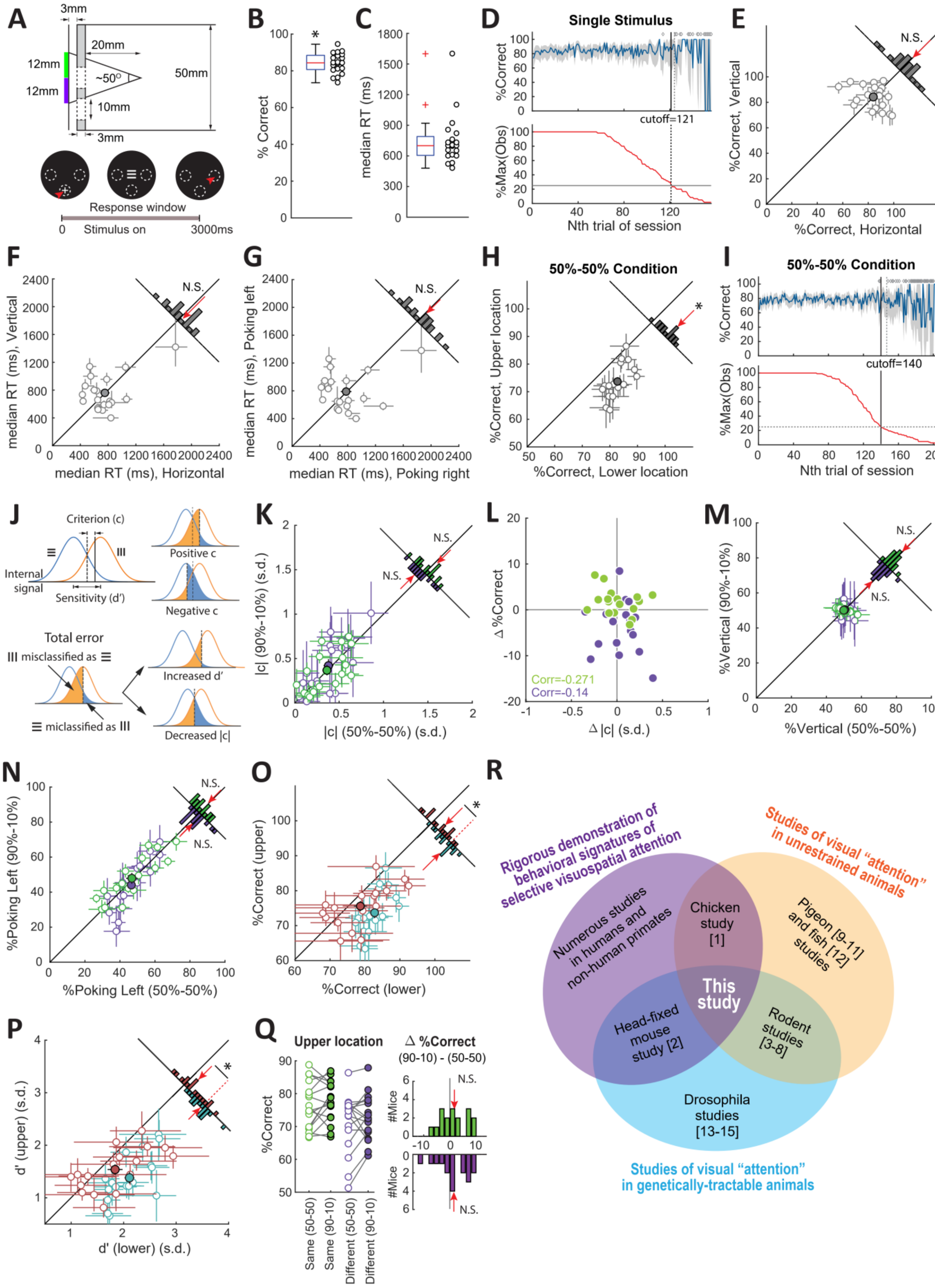
A
3mm
20mm
12mm
12mm
~50°
50mm
10mm
3mm
Response window
0
Stimulus on
3000ms
B
% Correct
*
C
median RT (ms)
D
Single Stimulus
%Correct
cutoff=121
%Max(Obs)
Nth trial of session
E
N.S.
%Correct, Vertical
%Correct, Horizontal
F
N.S.
median RT (ms), Vertical
median RT (ms), Horizontal
G
N.S.
median RT (ms), Poking left
median RT (ms), Poking right
H
50%-50% Condition
%Correct, Upper location
%Correct, Lower location
I
50%-50% Condition
%Correct
cutoff=140
%Max(Obs)
Nth trial of session
J
Criterion (c)
Internal signal
Sensitivity (d')
Positive c
Negative c
Total error
III misclassified as ≡
≡ miclassified as III
Increased d'
Decreased |c|
K
N.S.
N.S.
|c| (90%-10%) (s.d.)
|c| (50%-50%) (s.d.)
L
Δ %Correct
Δ |c| (s.d.)
Corr=-0.271
Corr=-0.14
M
N.S.
N.S.
%Vertical (90%-10%)
%Vertical (50%-50%)
N
N.S.
N.S.
%Poking Left (90%-10%)
%Poking Left (50%-50%)
O
*
%Correct (upper)
%Correct (lower)
P
*
d' (upper) (s.d.)
d' (lower) (s.d.)
Q
Upper location
%Correct
Same (50-50)
Same (90-10)
Different (50-50)
Different (90-10)
Δ %Correct
(90-10) - (50-50)
N.S.
#Mice
#Mice
N.S.
R
Rigorous demonstration of behavioral signatures of selective visuospatial attention
Studies of visual "attention" in unrestrained animals
Numerous studies in humans and non-human primates
Chicken study [1]
Pigeon [9-11] and fish [12] studies
This study
Head-fixed mouse study [2]
Rodent studies [3-8]
Drosophila studies [13-15]
Studies of visual "attention" in genetically-tractable animals

Supplementary Figure 1. Related to Figure 1. Single-stimulus discrimination task and spatial probability task: trial exclusion, location asymmetry, signal detection theory (SDT), and lack of sensory or motor biases.

(A-G) Single-stimulus visual discrimination task: Freely behaving mice learn feature-response association (n=20 mice). **(A)** Top row: Lateral view of the schematic experimental setup showing the relative position of the touchscreen (leftmost vertical line), the plexiglass mask (grey-filled vertical bar), and the tube within which mice move (50 mm diameter); the plexiglass mask is positioned 3 mm in front of the touchscreen. Dashed lines indicate the central response hole (lower dashed lines), and left/right response holes (upper dashed lines; 10 mm diameter). For single-stimulus discrimination, the center of the stimulus is aligned with the center of left/right response holes in elevation, and with the central hole in azimuth (see Bottom-row). For the spatial probability task, the upper (green) and lower (purple) locations of the stimulus are indicated as colored bars (see also Fig. 1C) For the flanker task, a 10 pixels (2 mm) spacing was introduced between the target at lower location and the flanker at upper location (see also Fig. 4A). The 60 pixels x 60 pixels (12mm x 12mm) stimulus subtends a visual angle of 25º when viewed from 20 mm front of the plexiglass mask. Bottom-row: Screen-shots of the display at different stages in a single-stimulus trial. Trials began with a nose-touch (red arrowhead) on a zeroing-cross presented within the lower, central hole. A single oriented grating ('target') was presented immediately after trial initiation (Methods). Mice were rewarded if they responded to the target per the following rule: vertical target grating → nose-touch in left response port; horizontal target grating → nose-touch in right response port. **(B)** Response accuracy; median ± 95% C.I. = 84.3 ± 2.8%. **(C)** Median reaction time (RT); median ± 95% C.I. = 700 ± 220 ms. Each circle denotes data from one mouse, calculated after pooling its trials from all behavioral sessions (Methods). Box-plot summarizes the distribution with the central mark being the median, the bottom/top edge of the box indicating the $25^{th}/75^{th}$ percentiles, and whiskers extending to the most extreme data points not considered outliers; asterisk: statistically significant from chance level ($p<0.001$, two-sided signed-rank test); red cross denotes outliers. **(D)** Identification of trials (late in sessions) that corresponded to animals being poorly engaged in the task (Methods). Top panel: Time course of overall response accuracy across mice as a function of trial number within sessions. Accuracy obtained from trials pooled across all mice and sessions, and computed as a function of trial number within session (blue; Methods). Grey shading: bootstrapped estimates of the 95% confidence interval of the accuracy (gray; Methods). Open diamonds: accuracy not significantly different from chance for corresponding trial. Dashed vertical line: first trial at which the accuracy was not different from chance (50%), and stayed indistinguishable from chance for 3/5 of the next 5 trials (Methods). Data show increased variability and worse performance towards the end of sessions. Bottom panel: Number of actual observations across mice for each trial number, as a percentage of the maximal number of possible observations (Σmice*sessions), plotted as a function of trial number within session (red). Data show drop in the number of observations available to reliably assess performance towards the end of sessions. Based on these data, all trials above 121 (black vertical line) of each behavioral session of this task were dropped from analysis (Methods). Results in panels B, C, and E-G are based on data from trials 1-121 from each behavioral session. **(E-F)** Scatter plot of response accuracy (E) and median reaction time (F) when the target was a horizontal grating versus when it was a vertical grating. Each harrow circle represents individual mouse: mean ± 95% C.I.; Filled circle: group mean ± S.E.M. Inset: Distribution of difference in performance between the horizonal and vertical target. Red arrow: median; $p=0.97$ (E); $p=0.97$ (F), two-tailed Wilcoxon signed-rank test (against difference = 0); N.S.: not statistically significant. **(G)** Scatter plot of median RT to nose-touches to right versus left response holes ($p=0.9$). Conventions as in E.

(H-Q) Spatial probability task (n=17 mice). **(H)** Scatter plot comparing response accuracy (%-correct) at the upper location versus the lower location in the (baseline) 50-50 condition. Conventions as in E. Median change = -10.9%, $p<0.001$, two-tailed signed rank test (against change = 0). Asterisk: $p<0.05$. **(I)** Identification of trials (late in sessions) that corresponded to animals being poorly engaged in the task (Methods; conventions as in D, above). Top panel: Overall response accuracy versus trial number, obtained from trials pooled across all mice and locations from 50-50 sessions. Bottom panel: Number of actual observations across mice for each trial number, as a percentage of the maximal number of possible observations (Σmice*sessions). Based on these data, all trials

above 140 of each behavioral session of this task were dropped from analysis (Methods). Results in Figures 1 and 2 are based on data from trials 1-140 from each behavioral session. **(J)** Schematic of the signal detection theory (SDT) analysis. Upper row; left: SDT hypothesizes that the internal representation of vertical and horizontal stimuli can be reduced (projected) to a one-dimensional decision axis, on which they form two overlapping distributions (due to noise). A decision is made based on a criterion set by each individual animal: a stimulus whose representation falls above (or below) the criterion is judged as vertical (or horizontal), producing the appropriate behavioral response. A decision criterion (c) of 0, by definition, corresponds to optimal (unbiased) performance given the two distributions. For our 2-AFC task, we defined the decision criterion as the amount of deviation from an unbiased value for the following reason. Upper row; right: Because of the inherent symmetry of 2-AFC task design, positive criterion would increase errors in classification of vertical targets, but also slightly decrease errors in classification of horizontal targets, producing a net reduction in overall accuracy. Similarly, a negative criterion would increase errors in classification of horizontal targets, but also slightly decrease errors in classification of vertical targets, again producing a net decrease in overall accuracy. Therefore, when the two distributions are similar, a negative as well as a positive criterion of the same magnitude will produce the same overall reduction in discrimination accuracy, but a criterion of smaller absolute value would signal an overall improvement in performance. For this reason, we used the absolute value of c (|c|) to examine the effect of criterion change on response accuracy. Lower row: Based on theory, improved response accuracy can result from (1) increased d': when the two distributions become further separated; or (2) decreased |c|: when the decision criterion becomes less biased. **(K)** Scatter plot comparing the criterion in the 90u-10 versus 50-50 condition at the upper (green data, p=0.981) and lower (purple data, p=0.332) locations. Conventions as in E. **(L)** Change in response accuracy plotted against change in criterion at the upper (green, Pearson's correlation=-0.271, p=0.292) and lower (purple, Pearson's correlation=-0.14, p=0.592) locations. **(M)** Scatter plot comparing percentage of vertical stimulus presentations between the two conditions at the upper (green, p=0.554) and lower (purple, p=0.868) locations. Conventions as in E. **(N)** Scatter plot comparing percentage of left nose-touches between the two conditions at the upper (green, p=0.356) and lower (purple, p=0.435) locations. Conventions as in E. No significant change in both cases (M, N) indicates that differences in sensory input or motor biases cannot account for the effects in Fig. 1. **(O)** Scatter plot comparing response accuracy (% correct) at the upper versus lower locations in the 50-50 condition (teal, median difference between locations =-10.9%, upper minus lower) and 90u-10 condition (maroon, median difference=-4.4%). The net change (6.5%) caused by spatial attention is significant from zero (p=0.004, two-tailed signed rank test). Conventions as in E. Asterisk: p<0.05. **(P)** Scatter plot comparing perceptual sensitivity accuracy (d') at the upper versus lower locations in the 50-50 condition (teal, median difference=-0.74 s.d.) and 90u-10 condition (maroon, median difference=-0.17 s.d.). The net change (0.57 s.d.) caused by spatial attention is significant from zero (p=0.005, two-tailed signed rank test). Conventions as in E. Asterisk: p<0.05. **(Q)** Examination of sequential effects at the upper location. Left panel: For successive trials in which the target was presented at the upper location, plot of probability of a correct response on the second trial when the target was the same in both trials (green data), and when the target was different in the two trials (purple data). Each line denotes data from one mouse. Right panel: Distribution of change in probabilities (across n=17 mice) between the 90u-10 and 50-50 conditions; colors as in left panel (green, p=0.287; purple, p=0.246, two-tailed signed-rank test against change = 0). Red arrow: median; N.S.: not statistically significant. **(R)** Schematic showing the three goals accomplished uniquely by the current study: (a) the demonstration of primate-like visuospatial selective attention [1,2]; (b) in freely moving animals [3-12]; and (c) in a species that facilitates the use of diverse genetically-based tools for neural interrogation[13-15]. Source data are provided as a Source Data file

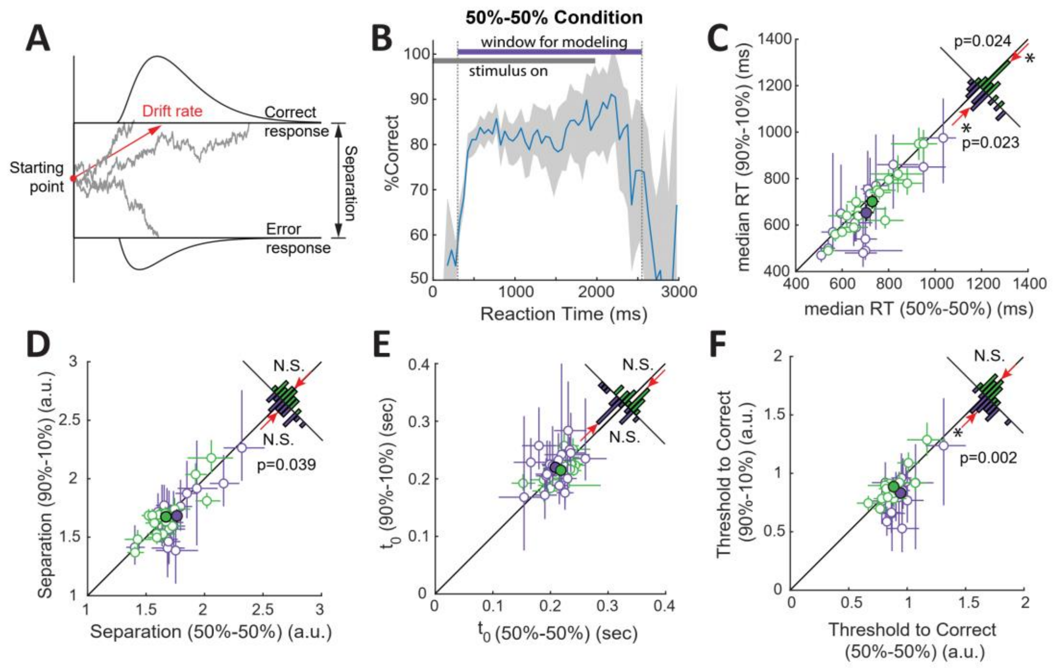

Supplementary Figure 2. Related to Figure 2. Spatial probability task: Drift diffusion modeling (n=17 mice) **(A)** Schematic diagram of the two-choice drift diffusion model. The model simulates a decision process from sensory stimulus presentation to the point of behavioral report, and attempts to account for the full distribution of observed RTs. It posits that upon stimulus presentation, sensory evidence flows in, causing a (hypothetical) decision variable to 'drift' either upwards (towards one choice boundary) or downwards (towards the other) depending on which choice the incoming evidence favors. Under uncertainty, the decision variable drifts in a stochastic (zig-zag) manner as sensory evidence accumulates, eventually crossing one of the decision boundaries and triggering the corresponding behavioral response. Here, we adopted a standard version of the model with four parameters: (i) drift rate, or the average rate of evidence accumulation, whose sign could be either positive (favoring response A) or negative (favoring response B); (ii) boundary separation, the distance by which the two decision boundaries are separated; (iii) starting point, which captures an initial bias towards one or the other choice (starting point = 0.5 indicates an unbiased decision maker), and (iv) a non-decisional constant (t_0), which accounts for net delay due to sensory encoding (before decisional process) and motor execution (after a decision has been made); not illustrated here. **(B)** Exclusion of trials with outlier RT values (inordinately short or long RTs) prior to diffusion modeling (Methods): Response accuracy plotted as a function of RT (binned into 50 ms bins). Here, accuracy was computed by first pooling trials across all mice, all sessions (of 50-50 conditions), and both locations, and then computing % correct from the trials within each RT bin. Gray shading: 95% C.I. obtained by bootstrapping. Based on this data, trials with RTs shorter than 300 ms or longer than 2550 ms, which exhibited accuracy that was not distinguishable from chance, were excluded from diffusion modeling. The same window was then applied to data of 90u-10 condition. **(C)** Outlier trial exclusion did not impact the effects on median RT reported in Fig. 2A (i.e., median RT was shorter in the 90u-10 condition at both locations). Green: upper location; purple: lower location. Each harrow circle represents individual mouse: mean ± 95% C.I.; Filled circle: group mean ± S.E.M. <u>Inset:</u> Distribution of difference in RT between two conditions. Red arrow: median; asterisk: $p<0.05$; N.S.: not statistically significant; two-tailed Wilcoxon signed-rank and HB test (against difference = 0). **(D-F)** Results from drift diffusion modeling: effect of spatial probability manipulation on the other two parameters of the model - boundary separation (D, green, p=0.723; purple, p=0.039), and non-decisional constant (t_0; E, green, p=0.332; purple, p=0.32). Conventions as in C. No systematic modulation of either parameter, at either location, by spatial probability. (F) Effect of spatial probability on 'threshold to correct responses' (a quantity computed as the difference between upper boundary and starting point; green, p=0.868; purple, p=0.002). Conventions as in C. Source data are provided as a Source Data file.

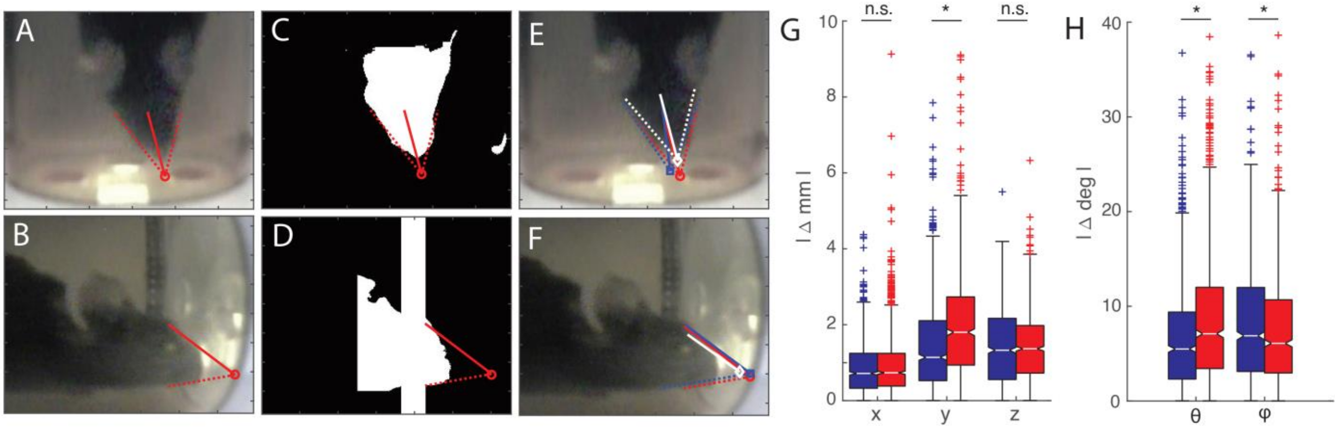

Supplementary Figure 3. Related to Figure 1, 2. Automated, marker-less tracking of the mouse head movement. **(A-B)** Example image frames from the top view (A) and the lateral view (B) videos of a mouse engaged in the spatial probability task; cropped just around the head. **(C-D)** Binarized versions of A and B, obtained using custom plug-in in FIJI (Methods). Dashed red lines: Estimated snout cone; red circle: Estimated position of the tip of the snout ('head') in each view; solid red line: Estimated orientation (angle) of the head in each view. Estimates were obtained using automated method (Methods; custom code in MATLAB). **(E-F)** Reproduction of A and B, but overlaid with manually estimated snout cones; white: human 1 (WKY); blue: human 2 (RP). **(G-H)** Quantitative comparison of head positions and angles estimated by automated and manual methods (n=1020 frames); results validate the automated method. Blue box plots: Distribution of absolute values of difference between estimates by two humans ('errors between humans'); red box plots: Distribution of absolute value of difference between estimates by the automatic method, and the mean of the estimates by the two humans ('errors by the automated method'). Box-plot summarizes the distribution with the central mark being the median, the bottom/top edge of the box indicating the 25th/75th percentiles, and whiskers extending to the most extreme data points not considered outliers. Asterisk: $p<0.05$; 'n.s.': not statistically significant, two-sided signed-rank test with HB correction (Methods). For x and z position estimates, the errors made by the automated method were not distinguishable, on average, from errors between humans (x, $p=0.205$; z, $p=0.535$). For estimates of y, θ, although the errors by the automated method (red) were statistically larger than errors between humans (y, $p<0.001$; θ, $p<0.001$), the magnitude of the differences are small. Median values for Δy: 1.14 mm (human-human) vs. 1.80 mm (auto-human); for Δθ: 5.49º vs. 7.10º. For φ, the error by the automated method was statistically smaller than the errors between humans ($p=0.023$), but this reduction was also small. Median values for Δφ: 6.89º (human-human) vs. 6.08º (auto-human). These data indicate that the estimates obtained by the automated method are not systematically less reliable than manual estimates. Source data are provided as a Source Data file.

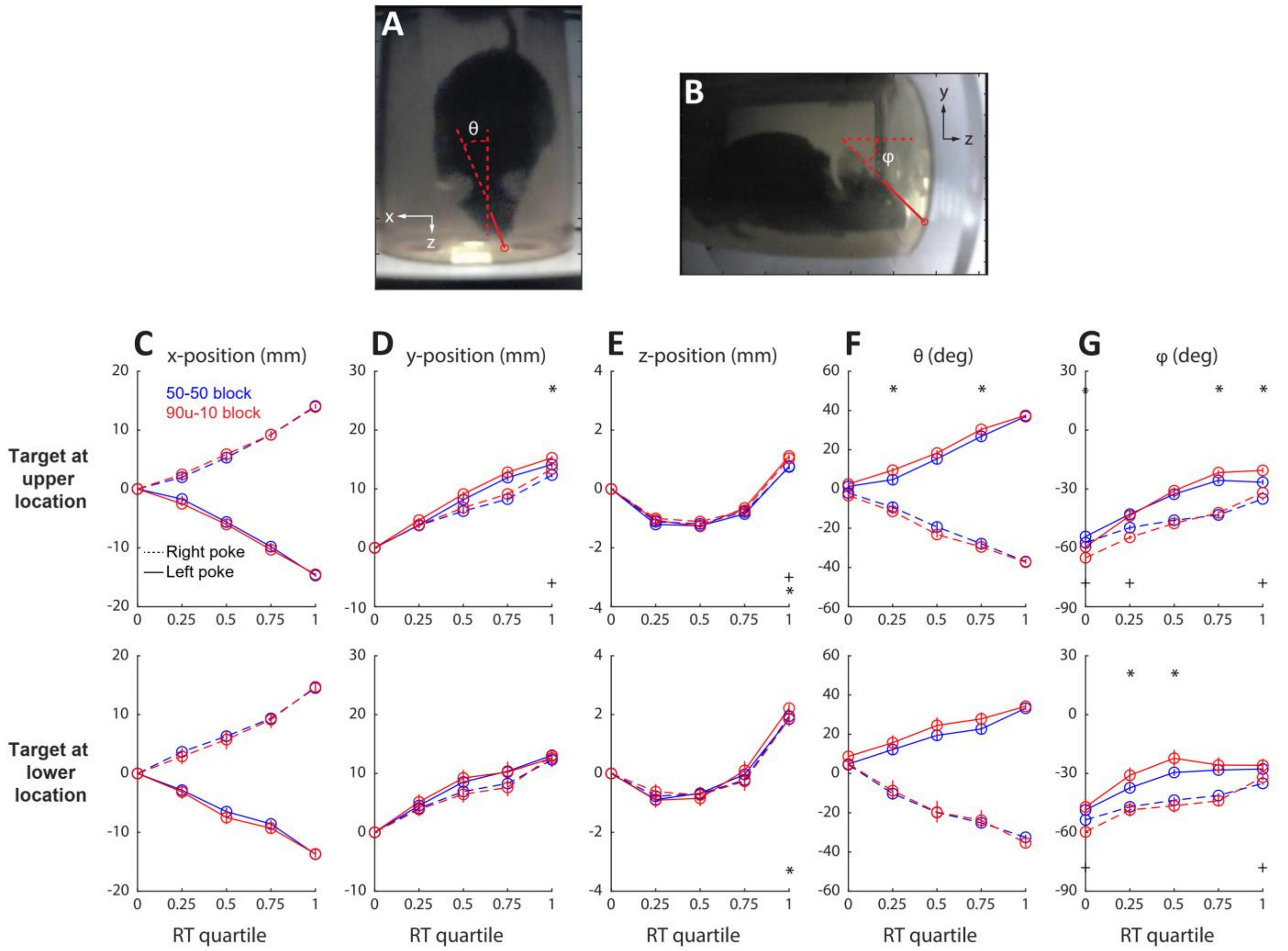

Supplementary Figure 4. Related to Figures 1, 2, and Supplementary Figure 3. Automated video-based tracking of mouse head position and orientation in 3D during spatial probability task. (A-B) Example image frames from the top view (A) and the lateral view (B) videos of a mouse engaged in the spatial probability task (Methods). Red circle: Estimated position of the tip of the snout ('head') in each view. Solid red line: Estimated orientation (angle) of the head in each view. The x- and z-positions of the snout tip, and the yaw angle (θ) were estimated from the top-view image, while the y-position and the pitch angle (φ) were estimated from the lateral-view image (Methods). The midpoint of the central response hole (i.e., the trail initiation hole) was set as the origin: [x,y,z]=[0,0,0]; leftward (from the mouse's perspective) yaw angles were positive, and downward pitch angles were negative. **(C-G)** Estimates of the head positions (in 3D) as well as head angles (yaw and pitch) during head movement from trial initiation (nose touch at zeroing cross) to trial completion (nose touch at left or right response hole for behavioral report). Data are plotted by RT quartiles: 0 – trial initiation, 0.25 – end of first quartile of trial RT, 1– end of 4th quartile of trial RT, i.e., trial completion. In blue: estimates from trials in the 50-50 blocks, in red: from trials in the 90u-10 blocks. Data for each condition were obtained from n=7 mice, 3 sessions of each condition per mouse (Methods); shown are mean (circles) and 95% CI of the mean (vertical line; sometimes smaller than the circle), estimated by bootstrapping at each RT quantile. Top (bottom) row: data from trials when the target was presented at the upper (lower) locations. Solid (dashed) lines: trials involving nose touches into the left (right) response holes. Asterisk/cross: $p<0.05$ for comparison between 90u-10 and 50-50 conditions (asterisk for left-poking trials; cross for right-poking trials, respectively); p-values were obtained by permutation test (two-sided) followed by correction for multiple comparisons (Methods). Any statistically significant differences in the head position and angle were small in magnitude (~5% of the whole range of movement). Moreover, these differences occurred primarily at the very end of the movement (C-E) – long after

when the decision about which direction to move needed to be made. Although the yaw (F) and pitch angle (G) showed significant differences also at the start of the trial, these changes as well could not account for the observed behavioral results. This was because the yaw angle (θ) was marginally more positive (greater leftward tilt) early in pokes to the left (but not right), thereby having no predictable impact on discrimination performance at the upper location. Similarly, the pitch angle (φ) was marginally more negative (greater downward tilt) at trial initiation, thereby also having no predictable contribution to improving discrimination performance at the upper location. Source data are provided as a Source Data file.

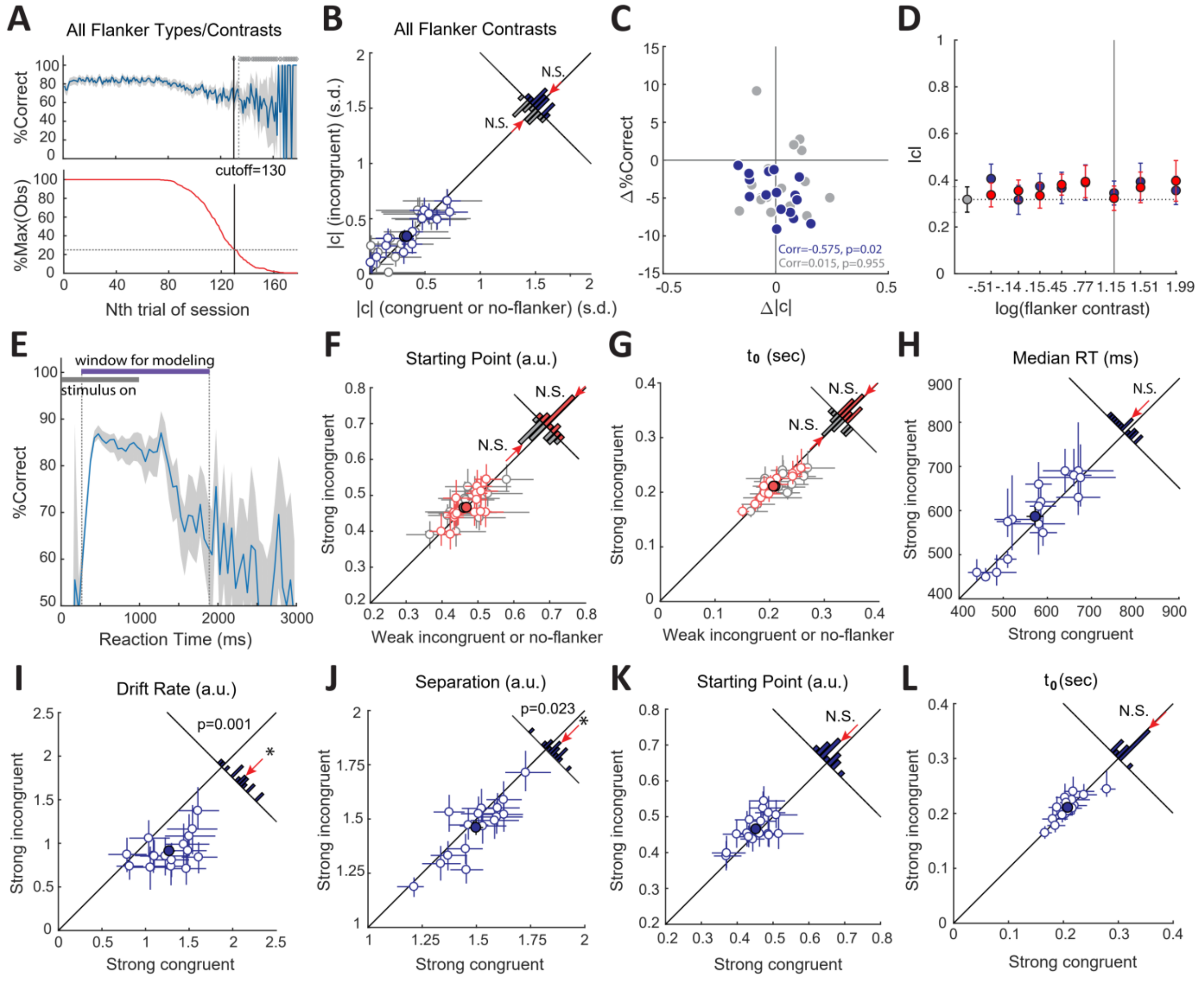

Supplementary Figure 5. Related to Figures 3 and 4. Flanker task: trial exclusion criterion, and effect of flanker contrast on RTs and parameters of the drift diffusion model (n=16 mice)

(A) Identification of trials (late in sessions) that corresponded to animals being poorly engaged in the task. Top panel: time course of overall response accuracy across mice as a function of trial number within session, obtained from trials pooled across all mice, flanker conditions and contrasts (Methods; conventions as in Supplementary Figure 1D). Bottom panel: number of actual observations across mice for each trial number, plotted as a percentage of the maximal number of possible observations (Σ mice*sessions). Based on these data, all trials above 130 of each behavioral session of this task were dropped from analysis (Methods). Results in Figures 3 and 4 are based on data from trials 1-130 from each behavioral session. **(B)** Scatter plot comparing response criterion (|c|) between the flanker conditions. Each harrow circle represents individual mouse: mean ± 95% C.I.; Filled circle: group mean ± S.E.M. Inset: Distribution of difference in performance between two flanker conditions. Blue: incongruent trials versus congruent trials (p=1); grey: incongruent trials versus no-flanker trials (p=0.438); two-sided signed-rank test (against difference = 0). Red arrow: median; N.S.: not statistically significant (p>0.05). **(C)** Plots of changes in response accuracy against changes in criterion. Colors as in B. Corr = Pearson's correlation. **(D)** Plot of decision criterion (|c|) as a function of flanker contrast (2-way ANOVA, effect of congruency, p=0.817; effect of flanker contrast, p=0.976; interaction, p=0.987). Data presented as group mean ± S.E.M. Gray vertical line: fixed contrast of the target. Gray data: No flanker; blue: congruent flanker; red: incongruent flanker. **(E)** Exclusion of trials with outlier RT values (inordinately short or long RTs) prior to

diffusion modeling (Methods); all conventions same as in Supplementary Figure 2B. Based on this data, trials with RTs shorter than 250 ms or longer than 1900 ms, which exhibited accuracy that was not distinguishable from chance, were excluded from diffusion modeling. **(F, G)** Drift diffusion modeling. Scatter plots comparing starting point (F), and non-decisional constant (t_0, G) in trials with strong versus weak incongruent flankers (red), and in trials with strong incongruent flankers versus no flanker (gray). Conventions are as in B. (F) red data, p=0.642; grey data, p=0.717. (G) red data, p=0.501; grey data, p=0.793. **(H)** Scatter plot comparing median RTs in trials with strong incongruent versus strong congruent flankers (p=0.171). Conventions are as in B. **(I-L)** Drift diffusion modeling. Scatter plots comparing drift rate (I, p=0.001), boundary separation (J, p=0.023), starting point (K, p=0.179) and non-decisional constant (L, p=0.605), in trials with strong incongruent versus strong congruent flankers. Conventions are as in B. Incongruent flankers produced a reduction in drift rate (I) as well as a reduction in boundary separation (J) as compared to the congruent trials, together explaining a lack of increase in overall RT. Asterisk: $p<0.05$, N.S.: not statistically significant; two-sided signed-rank test (against change = 0). Source data are provided as a Source Data file.

Supplementary References:

1 Sridharan, D., Ramamurthy, D. L., Schwarz, J. S. & Knudsen, E. I. Visuospatial selective attention in chickens. *Proceedings of the National Academy of Sciences of the United States of America* **111**, E2056-2065, doi:10.1073/pnas.1316824111 (2014).

2 Wang, L. & Krauzlis, R. J. Visual Selective Attention in Mice. *Current biology : CB* **28**, 676-685.e674, doi:10.1016/j.cub.2018.01.038 (2018).

3 Fizet, J., Cassel, J. C., Kelche, C. & Meunier, H. A review of the 5-Choice Serial Reaction Time (5-CSRT) task in different vertebrate models. *Neuroscience and biobehavioral reviews* **71**, 135-153, doi:10.1016/j.neubiorev.2016.08.027 (2016).

4 Bushnell, P. J. Overt orienting in the rat: parametric studies of cued detection of visual targets. *Behavioral neuroscience* **109**, 1095 (1995).

5 Ward, N. M. & Brown, V. J. Covert orienting of attention in the rat and the role of striatal dopamine. *Journal of Neuroscience* **16**, 3082-3088 (1996).

6 Weese, G. D., Phillips, J. M. & Brown, V. J. Attentional orienting is impaired by unilateral lesions of the thalamic reticular nucleus in the rat. *Journal of Neuroscience* **19**, 10135-10139 (1999).

7 Phillips, J. M., McAlonan, K., Robb, W. G. & Brown, V. J. Cholinergic neurotransmission influences covert orientation of visuospatial attention in the rat. *Psychopharmacology* **150**, 112-116 (2000).

8 Marote, C. F. O. & Xavier, G. F. Endogenous-like orienting of visual attention in rats. *Animal cognition* **14**, 535-544 (2011).

9 Reynolds, G. S. Attention in the Pigeon 1. *Journal of the experimental analysis of behavior* **4**, 203-208 (1961).

10 Vyazovska, O. V., Navarro, V. M. & Wasserman, E. A. Pigeons deploy selective attention to efficiently learn a stagewise multidimensional visual discrimination task. *Journal of Experimental Psychology: Animal Learning and Cognition* **44**, 162-167, doi:10.1037/xan0000168 (2018).

11 Shimp, C. P. & Friedrich, F. J. Behavioral and computational models of spatial attention. *Journal of Experimental Psychology: Animal Behavior Processes* **19**, 26 (1993).

12 Saban, W., Sekely, L., Klein, R. M. & Gabay, S. Endogenous orienting in the archer fish. *Proceedings of the National Academy of Sciences* **114**, 7577-7581 (2017).

13 Sareen, P., Wolf, R. & Heisenberg, M. Attracting the attention of a fly. *Proceedings of the National Academy of Sciences* **108**, 7230-7235 (2011).

14 Van Swinderen, B. Competing visual flicker reveals attention-like rivalry in the fly brain. *Frontiers in integrative neuroscience* **6**, 96 (2012).

15 Sun, Y. *et al.* Neural signatures of dynamic stimulus selection in Drosophila. *Nature neuroscience* **20**, 1104-1113 (2017).
