# Supplementary material for: Endogenous and exogenous control of visuospatial selective attention in freely behaving mice
Source: Nat Commun. 2020 Apr 24;11:1986. doi: 10.1038/s41467-020-15909-2 (PMC7181831; doi:10.1038/s41467-020-15909-2)
Supplement: Supplementary file 2 — Description of Additional Supplementary Information [file 41467_2020_15909_MOESM2_ESM.pdf]

Description of Additional Supplementary Files

File Name: Supplementary Movie 1
Description: Related to Figures 1 and 2. Video snippet showing mouse performing the 50-50 block of the spatial probability task.

File Name: Supplementary Movie 2
Description: Related to Figures 3 and 4. Video snippet showing mouse perform.
