# Supplementary material for: Endogenous and exogenous control of visuospatial selective attention in freely behaving mice
Source: Nat Commun. 2020 Apr 24;11:1986. doi: 10.1038/s41467-020-15909-2 (PMC7181831; doi:10.1038/s41467-020-15909-2)
Supplement: Supplementary file 5 — Reporting summary [file 41467_2020_15909_MOESM5_ESM.pdf]

nature research

Corresponding author(s): Shreesh P. Mysore
Last updated by author(s): 2020/03/17

Reporting Summary

Nature Research wishes to improve the reproducibility of the work that we publish. This form provides structure for consistency and transparency in reporting. For further information on Nature Research policies, see Authors & Referees and the Editorial Policy Checklist .

Statistics

For all statistical analyses, confirm that the following items are present in the figure legend, table legend, main text, or Methods section.

n/a	Confirmed	
☐	☒	The exact sample size (*n*) for each experimental group/condition, given as a discrete number and unit of measurement
☐	☒	A statement on whether measurements were taken from distinct samples or whether the same sample was measured repeatedly
☐	☒	The statistical test(s) used AND whether they are one- or two-sided<br>*Only common tests should be described solely by name; describe more complex techniques in the Methods section.*
☐	☒	A description of all covariates tested
☐	☒	A description of any assumptions or corrections, such as tests of normality and adjustment for multiple comparisons
☐	☒	A full description of the statistical parameters including central tendency (e.g. means) or other basic estimates (e.g. regression coefficient) AND variation (e.g. standard deviation) or associated estimates of uncertainty (e.g. confidence intervals)
☐	☒	For null hypothesis testing, the test statistic (e.g. *F*, *t*, *r*) with confidence intervals, effect sizes, degrees of freedom and *P* value noted<br>*Give P values as exact values whenever suitable.*
☒	☐	For Bayesian analysis, information on the choice of priors and Markov chain Monte Carlo settings
☒	☐	For hierarchical and complex designs, identification of the appropriate level for tests and full reporting of outcomes
☐	☒	Estimates of effect sizes (e.g. Cohen's *d*, Pearson's *r*), indicating how they were calculated

Our web collection on statistics for biologists contains articles on many of the points above.

Software and code

Policy information about availability of computer code

Data collection	Behavioral data collection was done using KLimbic control software (v1.3, Med Associates).
Data analysis	All data analysis were done in MATLAB (R2018b) and FIJI (ImageJ 1.52p). Custom head-tracking software is available upon request to the corresponding author. (This is indicated in the Code Availability section as well.)

For manuscripts utilizing custom algorithms or software that are central to the research but not yet described in published literature, software must be made available to editors/reviewers. We strongly encourage code deposition in a community repository (e.g. GitHub). See the Nature Research guidelines for submitting code & software for further information.

Data

Policy information about availability of data

All manuscripts must include a data availability statement. This statement should provide the following information, where applicable:

- Accession codes, unique identifiers, or web links for publicly available datasets
- A list of figures that have associated raw data
- A description of any restrictions on data availability

The source data have been provided as a source data file.

Field-specific reporting

Please select the one below that is the best fit for your research. If you are not sure, read the appropriate sections before making your selection.

☒ Life sciences ☐ Behavioural & social sciences ☐ Ecological, evolutionary & environmental sciences

For a reference copy of the document with all sections, see nature.com/documents/nr-reporting-summary-flat.pdf

Life sciences study design

All studies must disclose on these points even when the disclosure is negative.

Sample size	No apriori sample size calculation was performed. A sample size of 16-17 mice was used for the two main behavioral tasks (see also below), consistent with sample sizes used in studies of this kind in the literature (for instance, Wang et al, 2018, Curr Biology). Each mouse was run (for multiple sessions) on all the conditions involved in a particular task.
Data exclusions	Data were excluded based on pre-established criteria commonly used in the literature. Spatial probability task: Data from any mice that were unable to learn the baseline task (<70% performance in the 50-50 condition) were excluded from analysis. Flanker task: Data from any mice that performed at overall accuracy of <70% correct (across all flanker contrasts) were excluded. Details in the manuscript.
Replication	For every experiment, data were collected from multiple mice (16-17), with multiple behavioral sessions per mouse (4-11 sessions/mouse for spatial probability task and 15-20 sessions per mouse for flanker task).
Randomization	All subjects' performance were compared to themselves across conditions (within-subject design and analysis)
Blinding	Blinding was not relevant because of within-subjects design

Reporting for specific materials, systems and methods

We require information from authors about some types of materials, experimental systems and methods used in many studies. Here, indicate whether each material, system or method listed is relevant to your study. If you are not sure if a list item applies to your research, read the appropriate section before selecting a response.

Materials & experimental systems

n/a	Involved in the study
☒	☐ Antibodies
☒	☐ Eukaryotic cell lines
☒	☐ Palaeontology
☐	☒ Animals and other organisms
☒	☐ Human research participants
☒	☐ Clinical data

Methods

n/a	Involved in the study
☒	☐ ChIP-seq
☒	☐ Flow cytometry
☒	☐ MRI-based neuroimaging

Animals and other organisms

Policy information about studies involving animals; ARRIVE guidelines recommended for reporting animal research

Laboratory animals	Male C57BL/6 mice from Jackson Laboratory
Wild animals	No wild animals were used in this study.
Field-collected samples	No field collected samples were used in this study.
Ethics oversight	All procedures followed the NIH guidelines and were approved by the Johns Hopkins University Animal Care and Use Committee (ACUC).

Note that full information on the approval of the study protocol must also be provided in the manuscript.
